# Sperm centriole assessment identifies male factor infertility in couples with unexplained infertility – a pilot study

**DOI:** 10.1016/j.ejcb.2022.151243

**Published:** 2022-05-27

**Authors:** Ankit Jaiswal, Tatiana Baliu-Souza, Katerina Turner, Nagalakshmi Nadiminty, Amarnath Rambhatla, Ashok Agarwal, Stephen A. Krawetz, James M. Dupree, Barbara Saltzman, Samantha B. Schon, Tomer Avidor-Reiss

**Affiliations:** a Department of Biological Sciences, University of Toledo, Toledo, OH 43606, USA; b Department of Urology, College of Medicine and Life Sciences, University of Toledo, Toledo, OH 43614, USA; c Department of Urology, Vattikuti Urology Institute, Henry Ford Health System, Detroit, MI 48202, USA; d American Center for Reproductive Medicine, Cleveland Clinic, Cleveland, OH 44195, USA; e Department of Obstetrics and Gynecology, Center for Molecular Medicine and Genetics, Wayne State University School of Medicine, Detroit, MI 48201, USA; f Center for Molecular Medicine and Genetics, C.S. Mott Center for Human Growth and Development, Wayne State University School of Medicine, Detroit, MI 48201, USA; g Department of Obstetrics and Gynecology, University of Michigan, Ann Arbor, MI 48109, USA; h School of Population Health, College of Health and Human Services, University of Toledo, Toledo, OH 43617, USA; i Division of Reproductive Endocrinology & Infertility, Department of Obstetrics and Gynecology, University of Michigan, Ann Arbor, MI 48109, USA

**Keywords:** Sperm, Centriole, Infertility

## Abstract

Unexplained infertility affects about one-third of infertile couples and is defined as the failure to identify the cause of infertility despite extensive evaluation of the male and female partners. Therefore, there is a need for a multiparametric approach to study sperm function. Recently, we developed a Fluorescence-Based Ratiometric Analysis of Sperm Centrioles (FRAC) assay to determine sperm centriole quality. Here, we perform a pilot study of sperm from 10 fertile men and 10 men in couples with unexplained infertility, using three centriolar biomarkers measured at three sperm locations from two sperm fractions, representing high and low sperm quality. We found that FRAC can identify men from couples with unexplained infertility as the likely source of infertility. Higher quality fractions from 10 fertile individuals were the reference population. All 180 studied FRAC values in the 10 fertile individuals fell within the reference population range. Eleven of the 180 studied FRAC values in the 10 infertile patients were outliers beyond the 95% confidence intervals (P = 0.0008). Three men with unexplained infertility had outlier FRAC values in their higher quality sperm fraction, while four had outlier FRAC values in their lower quality sperm fraction (3/10 and 4/10, P = 0.060 and P = 0.025, respectively), suggesting that these four individuals are infertile due, in part, to centriolar defects. We propose that a larger scale study should be performed to determine the ability of FRAC to identify male factor infertility and its potential contribution to sperm multiparametric analysis.

## 1. Introduction

Infertility, defined as the inability to conceive after 12 months of attempted conception (Medicine, 2020), affects about 12–20% of couples (Chandra et al., 2014; Mascarenhas et al., 2012; Thoma et al., 2013). In about one-third of these couples, infertility is due to a female factor, while in another third, a male factor is responsible. In the remaining one-third of couples, the cause may be due to a combination of the two (Pandruvada et al., 2021; Ray et al., 2012). When the medical evaluations of the male and female partners do not identify an etiology from either partner, the couple is diagnosed with “unexplained couple infertility” or “unexplained infertility.” Unexplained infertility is defined as the absence of identifiable causes for infertility using currently available testing (Moghissi and Wallach, 1983, Zegers-Hochschild et al., 2017). The apparent lack of information as to whether male or female deficiency underlies the condition limits treatment options.

Current male fertility examination is based on a patient history review, physical and hormonal examinations, semen analysis, and in rare cases, testing for DNA fragmentation and seminal oxidative stress (Boitrelle et al., 2021). Semen analysis provides general information, such as sperm number/concentration, motility, and morphology. This analysis looks at only some of the properties of the sperm and, in general, does not strongly correlate with a man’s fertility potential (Wang and Swerdloff, 2014). It has been suggested that undetected male factors could be identified by analyzing additional features that are essential for sperm function, such as RNA, proteins, and cellular structures (e.g., centrioles); this type of analysis is known as advanced semen analysis (Boitrelle et al., 2021; Jodar et al., 2015; Pandruvada et al., 2021; Patel et al., 2019a).

The extent to which various advanced sperm features (i.e., sperm cellular functions) are affected in unexplained infertility is unclear (Majzoub et al., 2019; Ostermeier et al., 2004; Tahmasbpour Marzouni et al., 2022). Multiparametric sperm analysis may be useful in two potential scenarios: (1) only one sperm feature may be involved in some infertile men, requiring a multiparametric sperm analysis as a way to screen the various sperm features and identify the affected feature; or (2) more than one sperm feature may be involved in other infertile men, requiring a multiparametric sperm analysis to obtain a complete picture of the sperm deficiency and identify the primary cause of infertility. As such, a multiparametric sperm analysis that includes an array of tests of distinct sperm features should provide a more reliable treatment direction and a more accurate prediction of treatment success. Furthermore, a multiparametric sperm analysis could provide psychological relief to an affected couple by determining the obstacle to a healthy pregnancy and, in the case of unexplained infertility, identifying either the male or the female as infertile, more effectively directing treatment. Finally, this knowledge can provide patients with the information necessary to seek out and join more specific advocacy groups that offer networks of support and updated information on the latest research.

One of the least tested sperm features are the centrioles (Avidor-Reiss et al., 2020), which function in the sperm during swimming toward the egg (Fishman et al., 2018; Khanal et al., 2021; Leung et al., 2021) and form the embryo’s first centrosomes (Amargant et al., 2021; Cavazza et al., 2021; Kai et al., 2021; Schatten and Sun, 2011; Terada et al., 2010). Centrioles are affected in some infertile men with abnormal sperm morphology (Chemes, 2012; Moretti et al., 2016; Nanassy and Carrell, 2008; Sha et al., 2017; Turner et al., 2021) and were found to be compromised in some cases of unexplained infertility in studies using transmission electron microscopy (Garanina et al., 2019).

The sperm centrioles form the two centrosomes of the zygotes. These centrosomes are needed for syngamy, bringing the male and female pronuclei together, and polarizing their genomes (Cavazza et al., 2021). Defects in this process results in lagging chromosomes during zygote cleavage, early embryo aneuploidy, and death resulting in miscarriages (Avidor-Reiss et al., 2022). Since sperm centriole defects can be present without significant consequence on sperm number, morphology, or shape, centriole defects would go undetected with current traditional semen analysis (Garanina et al., 2019).

**Fig. 1.**
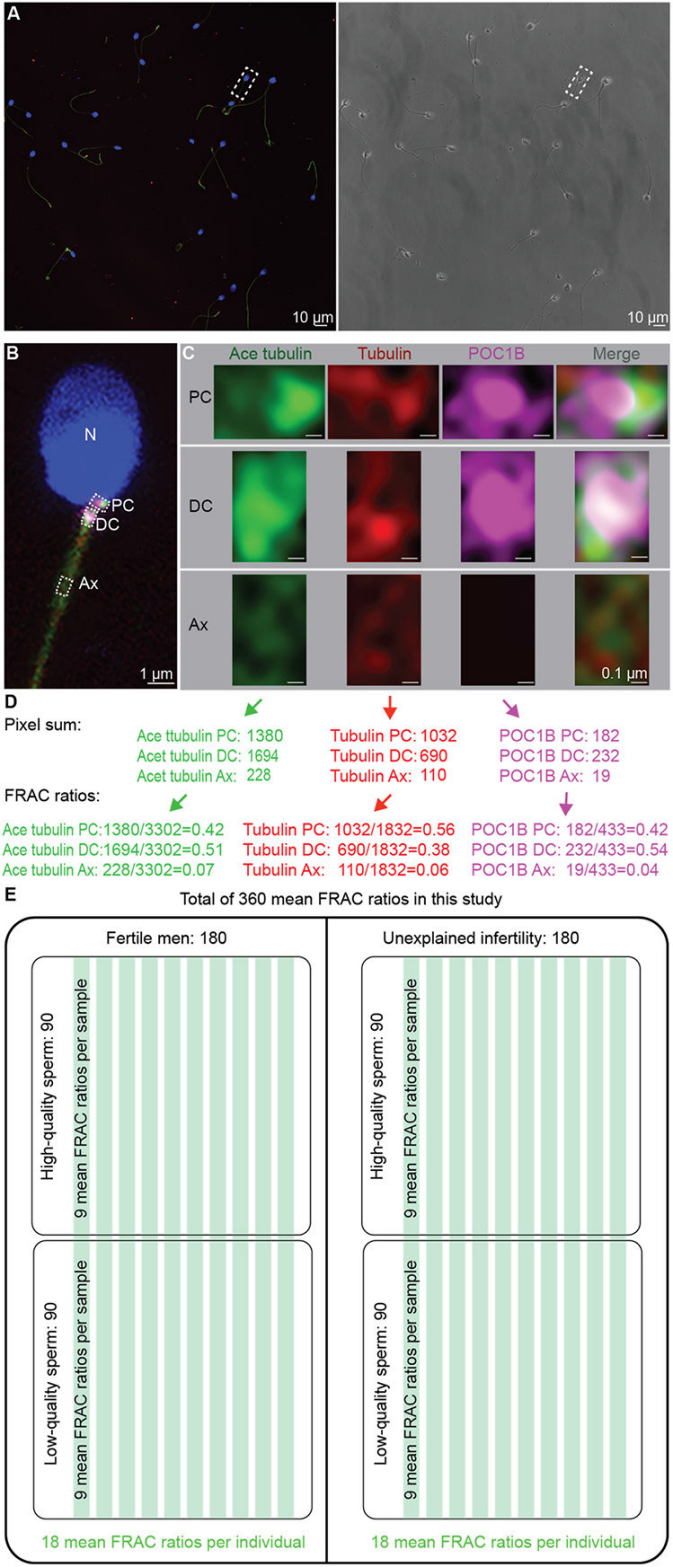
Quantitative imaging of sperm centriolar markers using the FRAC. (A) Examples of fluorescence and phase images of a group of high-quality spermatozoa. (B) Zoomed image of one sperm from panel A. Nucleus (N). Three boxes indicate the approximate location of the proximal centriole (PC), distal centriole (DC), and axoneme (Ax) labeled by the three different markers. (C) Zoomed image of the PC, DC, and Ax of the sperm from panel B labeled with anti-acetylated tubulin (Ace tubulin), anti-tubulin and anti-POC1B antibodies (Scale bar 0.1 μm). (D) Example of FRAC ratio calculation for a single sperm cell. (E) Overview of samples and groupings in the study indicating the number of mean FRAC ratios in each group. Each green line indicates a studied man.

Centriolar biomarkers can be structural proteins or post-translational modifications. Eleven biomarker proteins and one post-translational modification are known to be found in both the PC and DC: Tubulin, CETEN1/2, POC1B, POC5, CEP63, CEP90, CPAP, FAM161A, WDR90, WDR62, NEK9, and Acetylated tubulin (Amargant et al., 2021; Fishman et al., 2018; Khanal et al., 2021). Here, we analyzed three biomarkers (tubulin, POC1B, and acetylated tubulin), each of which was quantified in three locations (proximal centriole, PC; distal centriole, DC; axoneme, Ax), for a total of nine FRAC ratios per sperm cell (Fig. 1). Tubulin and Proteome of the Centriole 1B (POC1B) are structural components of the centriole, and tubulin is also a structural component of the axoneme (the sperm tail core structure). Tubulin is a heterodimer complex that polymerizes to form the centriole and axoneme wall (Avidor-Reiss and Gopalakrishnan, 2013; Winey and O’Toole, 2014). POC1B is an evolutionarily conserved centriolar protein that forms a luminal scaffold structure in canonical centrioles and novel rod structures in the sperm atypical distal centriole (DC) and is essential for centriole stability (Fishman et al., 2017; Keller et al., 2009; Khire et al., 2016; Le Guennec et al., 2020; Pearson et al., 2009). Both tubulin and POC1B were useful in identifying centriole defects in morphologically abnormal sperm using the FRAC method, and therefore were also used in this study (Turner et al., 2021).

Acetylated tubulin is a post-translational modification of tubulin (Piperno and Fuller, 1985) that is thought to increase microtubule elasticity and is associated with the stability of microtubules, such as those found in the centriole and axoneme (Guichard et al., 2021; Janke and Magiera, 2020; Nekooki-Machida and Hagiwara, 2020; Portran et al., 2017). It was reported that the total pool of acetylated α-tubulin is reduced in individuals with poor sperm motility (Bhagwat et al., 2014). Since most spermatozoan tubulin is found in the axoneme, this reduction likely reflects the situation in the axoneme. The precise relationship between the microtubules and POC1B (aka WDR51B) rod structures in the DC is unknown, though POC1B likely serves as scaffolding for the DC microtubules (Avidor-Reiss et al., 2019; Avidor-Reiss and Turner, 2019). Sperm centrioles are acetylated, possibly because they are part of a dynamic basal complex that is under large mechanical stress (Khanal et al., 2021). Therefore, we decided to use acetylated tubulin as a biomarker in this study.

We report that analyzing sperm centriolar biomarkers using fluorescence microscopy can identify male factor infertility in previously unexplained infertile couples. This suggests that analyzing sperm centrioles should be included in a multiparametric analysis of the sperm.

## 2. Methods

As part of a consortium, sperm samples from 20 individuals were analyzed. Semen analysis was performed to determine whether the basic properties of the 10 fertile and 10 infertile men were similar. Each sample was separated into two fractions (high- and low-quality sperm) using differential gradient centrifugation. Sperm were stained with fluorescence-tagged antibodies and imaged using a fluorescence microscope for FRAC analysis. Then, fluorescence intensity was quantified at three locations in each sperm, and the data was used to generate FRAC ratios. General basic tools were used to calculate mean FRAC ratios and 95% confidence FRAC ratios for each sample. The reference distribution was calculated from fertile high-quality sperm. Finally, the intraclass correlation coefficient (ICC) was used to determine the reliability of the quantification step. Because we analyzed three proteins (acetylated tubulin, tubulin, POC1B), at each of three locations (PC, DC, Ax), we generated nine FRAC ratio variables in total (i.e., acetylated tubulin PC, acetylated tubulin DC, acetylated tubulin Ax, tubulin PC, tubulin DC, tubulin Ax, POC1B PC, POC1B DC, and POC1B Ax) (Fig. 1). Samples with one or more 95% confidence FRAC ratios outside the reference range for any of the FRAC ratios were considered outliers.

### 2.1. Consortium and smart IRB

In November 2018, we formed the Collaborative Program for Translating Basic Research to Clinical Trial in Idiopathic Infertility (CPTBR) consortium, which aims to utilize multiparametric sperm analysis to study male infertility. This consortium utilizes semen samples collected as part of the Reproductive Subject Registry and Sample Repository (RSRSR) at the University of Michigan (UM IRB#-HUM00125627). The Institutional Review Board (IRB) at the University of Toledo approved this study (UT IRB#300364; initial approval 10/11/2019; PI: Tomer Avidor-Reiss). The remaining members of the consortium agreed to rely on the approval of the study by UT by utilizing a SMART IRB Reliance system (https://smartirb.org/reliance/; SMART IRB ID:2683; initial approval 4/11/2020). The University of Toledo IRB reviewed the SMART IRB, and the other members rely on the UT IRB for compliance with human subject research protections.

### 2.2. Eligibility criteria for participants

Semen samples were obtained from the University of Michigan RSRSR (Schon et al., 2021). The protocols and procedures of RSRSR have been approved by the Institutional Review Board of the University of Michigan Medical School (IRBMED), registered under IDHUM00125627. For this study, 20 de-identified samples were obtained from RSRSR for analysis. 10 samples were obtained from couples with unexplained infertility, defined as documentation of a normal semen analysis (WHO, 2010) and female partner with normal ovarian reserve (AMH ≥ 1.0 ng/mL, FSH <12mIU/mL), normal tubal evaluation by hysterosalpingogram or saline-infusion sonogram, normal menstrual cycles, and no other documented cause of infertility. 10 samples were also obtained from fertile men (prior reported history of at least one pregnancy, normal semen analysis, and no reported history of hormonal treatments). Subject age of both partners was also available for analysis. Within each cohort, we examined the means, standard deviations, and ranges of sperm count, volume, % normal morphology, and % motility using Microsoft Excel AVERAGE and STDEV functions.

Semen samples were produced by masturbation and ejaculated into containers in the privacy of a clinic room. The ejaculates were allowed to liquefy for at least 30 min at 37 °C. Semen analysis was performed according to WHO guidelines, which include information on assessing semen volume, sperm count, motility, and morphology; this information was used to determine sperm infertility phenotypes (WHO, 2010). Samples were separated into seminal and sperm fractions, cryopreserved, and stored in liquid nitrogen as previously described (Schon et al., 2021). Whether cryopreservation effects FRAC analysis outcomes is currently unknown. FRAC analysis was performed in 2021–2022 and was ended when all 20 originally planned samples were analyzed. No harm or unintended effects to patients were observed.

### 2.3. Differential gradient centrifugation, washing, attachment, and fixation

At the University of Toledo, semen samples were separated into interface and pellet sperm fractions using differential gradient centrifugation following manufacturer instructions (PureSperm). In summary, frozen samples, Medium 199 media (Sigma-Aldrich, M7528), and all necessary PureSperm® media were brought to 37 °C. Then, 1.0 mL of PureSperm® 80% was placed into a conical tube, labeled “pellet.” Next, 1.0 mL of PureSperm® 40% (Nidacon, PS40–100) was pipetted on top of the lower phase. The thawed semen sample (about 500 μL) was then pipetted on top of the upper phase. The conical tube was centrifuged for 20 min at 400 x g. Following centrifugation, the middle layer (about 500 μL) was removed and placed into a second tube, labeled “interface.” Remaining liquid was removed from the pellet tube without disturbing the pellet and was discarded appropriately. Both samples were washed with 2.0 mL PureSperm® Wash (Nidaon, PSW-100) media and were resuspended by pipette. Tubes were then centrifuged for eight minutes at 250 x g, after which supernatant (about 1900 μL) was removed from both tubes without disturbing the pellet. Both pellets were resuspended in 100 μL of Medium 199 media (Sigma-Aldrich, M7528). About 10 μL of both pellet and interface samples were pipetted onto a slide. Samples were visualized using light microscopy at 100X magnification and were diluted as necessary with Medium 199 (Sigma-Aldrich, M7528) media to ensure optimal concentration and distribution of sperm on slides. The optimal concentration of sperm was 20–30 sperm per 245 μm^2^, and the optimal distribution minimized sperm overlap with each other. Samples were then evenly aliquoted between 15 glass slides (Azer Scientific, EMS200A+) per pellet and interface samples, approximately 10.0 μL per aliquot. Glass cover slips (VWR, 48366–205) were then placed over each aliquot. Slides were then snap-frozen in a liquid nitrogen container and stored in a liquid nitrogen tank until needed.

### 2.4. Sperm staining

Each sample fraction/population was analyzed three times independently. Sample slides were retrieved from liquid nitrogen, and the coverslip was removed with forceps. Sample slides were fixed in prechilled methanol in a slotted glass Coplin jar at − 20 °C for five minutes. Slides were then washed with 1X PBS in a slotted glass Coplin jar for one minute, followed by permeabilization with 0.3% Triton X-100 (Sigma-Aldrich, 9002–93–1) in PBS in a slotted glass Coplin jar for one hour, and finally blocked with 1% BSA (CHEM-IMPEX INT’L, 00535) in PBS with 0.3% Triton X-100 in a slotted glass Coplin jar for 30 min. 200 μL of primary antibodies diluted in PBS with 1% BSA and 0.3% Triton X-100 were applied to each slide, which were then covered with Parafilm (Bemis^™^, 13–374–12) and incubated overnight at 4 °C (Table 1).

On the following day, slides were washed three times for five minutes per wash in PBS with 0.3% Triton X-100 in a slotted glass Coplin jar. Subsequently, slides were incubated with secondary antibodies by applying 200 μL of secondary antibodies diluted in PBS with 1% BSA and 0.3% Triton X-100 to each slide, which were then covered with Parafilm (Bemis^™^, 13–374–12) and incubated at room temperature for one hour. Slides were then washed three times for five minutes per wash in PBS with 0.3% Triton X-100 in a slotted glass Coplin jar. Samples were then washed three times for five minutes per wash in PBS in a slotted glass Coplin jar. One drop of Fluoroshield with DAPI (Sigma-Aldrich, F6057) was added to the middle of each slide, glass coverslips (Thermo Scientific, 16940) were placed over each sample, and each coverslip was sealed with clear nail polish. Slides were kept at − 20 °C until analysis.

### 2.5. Confocal microscopy

Slides were visualized using a Leica SP8 confocal microscope in photon counting mode using an HC PL APO CS2 63x/1.40 OIL lens, 10% gain, 4096 × 4096 pixels (245 μM x 245 μM) format, 0.75 zoom factor, 2.0 frame accumulation, and rotation set at 90.00 (Fig. 1A). Fluorescence signal was collected using four sequences. Sequence one produced two images: DNA and phase-like. To capture DNA staining via DAPI, we activated a 410 nm (UV) laser at 0.1% power. The absorption spectrum was set to cover 412–474 nm via HyD1 detector and was assigned blue. To create a phase-like picture, PMT Trans was set to ON with a gain of 250, and the fluoro turret was set to Scan-PH (Phase). To capture acetylated tubulin staining via ALEXA 488 (sequence two), we activated a 488 nm laser set at 4% power. The absorption spectrum was set to cover 506–545 nm via HyD3 detector and was assigned green. To capture tubulin staining via ALEXA 555, we activated a 561 nm laser set at 4% power (sequence three). The absorption spectrum was set to cover 563–615 nm via HyD3 detector and was assigned red. To capture POC1B staining via ALEXA 647, we activated a 633 nm laser set at 4% power (sequence four). The absorption spectrum was set to cover 651–695 nm via HyD4 detector and was assigned magenta.

We collected multiple (10–20) Z-sections of 0.3 μM thickness from the top of the highest sperm to the bottom of the lowest sperm. In all experiments, the biomarker intensity signal was not saturated in the sperm PC, DC, nor Ax, and the photon count per pixel in the images ranged between 0 and 42 (Fig. 1C).

Fluorescence microscopy is complicated by many factors, which introduces high variability to the measured signal intensity (Dunn et al., 1994). Therefore, studies requiring quantitative fluorescence intensity measurements must consider this complication of variability by measuring the illumination power density (Montero Llopis et al., 2021). The advantage of the FRAC method is that it compares data normalize by a local ratio; therefore, the FRAC method is relatively insensitive to fluctuation in fluorescence intensity measurements and illumination power density.

To measure the illumination power density of the laser of the confocal microscope, we used an optical power and energy meter (Newport 1918-C). The energy was measured by replacing one of the objectives with a silicon detector. The total incident energy over a period of 60 second was acquired at maximum available laser power (100% emission level selected in the software). Each measurement was repeated three times. We then calculated the illumination power by dividing the energy by 60 seconds. Illumination power density was calculated by dividing the power with the aperture area (which was ~50 mm^2^).

This measurement was carried out for each individual laser used for our experiment for three consecutive days. The mean illumination power density of the diode 405 LASOS laser (@405 nm), argon laser (@488 nm), DPSS 561 laser (@561 nm), and the HeNe 633 laser (@633 nm) were 17.7 ± 0.3 μw/cm^2^, 10.8 ± 0.2 μw/cm^2^, 9.6 ± 0.2 μw/cm^2^, and 7.1 ±0.1 μw/cm^2^, respectively. Overall, the deviation over 3 days was less than 2.5%. The detector was already pre-calibrated to account for the difference in sensitivity at each of the specific wavelength.

Imaging was conducted between January 2021 to August 2021.

### 2.6. Staining quantification

**Fig. 2.**
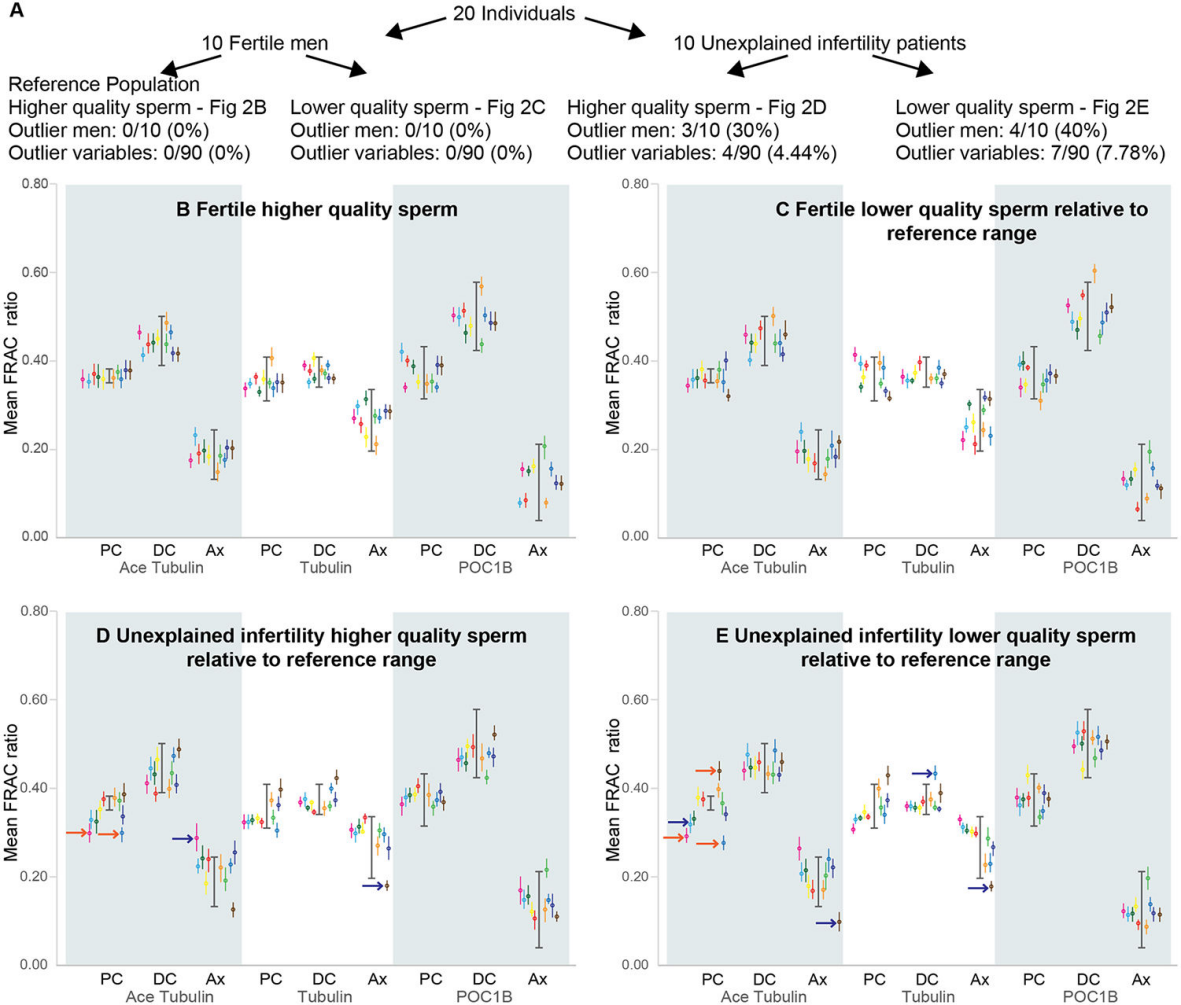
FRAC identifies suboptimal centrioles in unexplained infertility.

Quantification of the results, described in Fig. 2, was performed by one experienced rater that was blinded to the sperm sample identity using the Leica Application Suite X (LASX) program. For each image, composed of multiple Z planes, we generated a max projection that was used to identify the sperm and the regions of interests (ROIs). The intensity of staining for each marker in the acquired images was quantified in the PC, DC, and Ax of each sperm using a 0.5 × 0.75 μM rectangle ROI overlayed using the Draw Rectangle tool (Fig. 1B-C). In general, POC1B staining was more specific and was therefore used to guide rectangle placement. The first ROI rectangle was placed over the PC, according to the greatest intensity in the PC. The second ROI rectangle was placed over the DC, according to the greatest intensity in the DC. The PC and DC rectangles were moved and rotated in such a way as to include the greatest possible amount of signal while also strictly avoiding box overlap. The third ROI rectangle was placed 2 μm from the bottom border of the DC, along the Ax. The 2 μm distance between the axoneme rectangle and the DC rectangle was arbitrary as it serves mainly as a control. The only considerations were to have it sufficiently far from the centriole to avoid confusion between the axoneme and centrioles, but not too far to catch centriolar protein that leaked into the axoneme.

When the centrioles could not be identified easily using signals from acetylated tubulin, POC1B, and DAPI, the following steps were taken to improve visibility: (i) decreasing or increasing the intensity levels of acetylated tubulin, POC1B, and DAPI to better identify and pick the signal; (ii) viewing one signal at a time, while turning the other signals off; and (iii) using tubulin as an additional guide signal. When both proximal and distal centrioles were not visible: (i) the PC rectangle was placed where the axoneme meets the head and was centered horizontally to the head; and (ii) the DC rectangle was placed vertically along the axoneme, forming a “T” shape with the PC rectangle. These guidelines were also used to place a second rectangle when only one (PC or DC) rectangle could be placed according to the intensity signal. When the axoneme could not be identified easily with signals from acetylated tubulin, POC1B, and DAPI, the phase signal was used to improve visibility. When the axoneme was not visible via any signal at the location where the Ax rectangle should be placed, the sperm was not quantified. When the axoneme was visible only at locations further away from the appropriate location of the third box, the sperm was quantified using a straight line between the centrioles and this signal. Each sperm had to meet two criteria to be quantified: (i) the location of all three rectangles had to be free from noise and obstructions; and (ii) the axoneme had to be visible in the location where the third rectangle was to be placed. Sperm were not quantified when any of the following were observed: no head, two heads, tailless heads, two tails, headless tails, or more than two centrioles per sperm. Per sample, each sperm in view was measured, regardless of sperm phenotype, with the exception of sperm excluded by any of the criteria mentioned above. Quantification values were gathered from the pixel sum calculation of each channel–tubulin, acetylated tubulin, and POC1B–provided by the LASX program.

### 2.7. Fluorescence-based ratiometric analysis of sperm centrioles (FRAC) ratio calculation

Average repeated individual fluorescence values have a very high deviation because immunofluorescence signal intensity is sensitive to many factors, and changes dramatically even in the same studied slide. To overcome this, we normalize the signal intensity locally, i.e., we divide the value of an individual sperm marker by the sum of all three locations in the same individual sperm. This generates a ratio we called the FRAC ratio (Fig. 1D).

Pixel sum data was copied to an Excel spreadsheet. The pixel sum ratios (FRAC ratios) of all three biomarkers at each of the PC, DC, and Ax were then calculated for each sperm of each patient. Using tubulin at the PC as an example, the FRAC ratio was calculated by dividing the pixel sum of tubulin at the PC by the sum of tubulin pixel sums at the PC, DC, and Ax (Fig. 1D). For example, if the pixel sum of tubulin at the PC was 30 and the pixel sum of tubulin at the PC, plus DC, plus Ax was 100, then the FRAC ratio of tubulin at the PC would be 0.30. Following this, the mean and confidence interval of the FRAC ratios of all sperm analyzed in a sample fraction (~100) were calculated; this mean is known as the sample mean FRAC ratio. Separate calculations were done for the higher and lower quality sperm fractions of each individual. For the reference range, higher quality sperm of fertile individuals were used. The average mean FRAC ratio as well as standard deviation of the mean FRAC ratio (SD) were calculated for all 10 samples in the reference population. The mean of the mean FRAC ratio ± two standard deviations (SDs) were used as a reference range that identifies any sample with 95% probability of belonging to the reference population. Samples with mean FRAC ratio outside the reference range for any of the mean FRAC ratio variables were considered outliers.

To compare the four sperm populations, we counted the number of outlier mean FRAC ratios out of the 90 (each population was comprised of 10 individuals, each with 9 mean FRAC ratios) measured FRAC ratios. To compare the two groups (fertile versus unexplained infertility), we counted the number of outlier mean FRAC ratios out of the 180 (each group was comprised of 10 individuals, each individual had two populations of sperm - high- and low- quality, and each individual sample had 9 mean FRAC ratios) measured FRAC ratios.

### 2.8. Reliability

To gain insight into the reproducibility of the quantification step, we assessed rater performance by comparing their nine mean FRAC ratios in 10 individuals (Supplemental Fig. 1). Intra-sample reliability of the FRAC analysis was calculated by ICC (Koo and Li, 2016). Because we selected our raters from a large population of raters with similar characteristics (students with no prior experience), we used a “Two-Way Random-Effects” Model. This model allows us to generalize our reliability results to any raters who possess the same characteristics as the raters selected for the reliability study. As we used the measurement from a single rater as the basis of the actual measurement, we used the “Single Rater/Measurement” Type. Since we were concerned with different raters assigning the same score to the same subject, we used the “Absolute Agreement” Definition. Then, we compared the raters to each other. Based on the 95% confidence interval of the ICC estimate, values less than 0.5 are indicative of poor reliability, values between 0.5 and 0.75 are indicative of moderate reliability, values between 0.75 and 0.9 are indicative of good reliability, and values greater than 0.90 are indicative of excellent reliability.

### 2.9. Statistical analysis

Normal distribution was determined after calculating skewness and kurtosis using the functions SKEW and KURT in Excel. We also tested for normality (Shapiro-Wilk, D’Agostino-Pearson, Jarque-Bera, Cramer-von Mises, and Anderson-Darling) using https://www.gigacalculator.com/ calculators/normality-test-calculator.php (used on February 22, 2022). Number of sperm (N) was calculated using the function COUNT. A T-Test and F-Test were performed using the functions F.TEST and T.TEST in Excel. The T statistic, using the equation {R⇤SQRT(N- 2)}/{SQRT(1-R2)}, and the degrees of freedom were calculated by N-2 in Excel. 95% confidence intervals (AKA margins of error) of individual sample mean FRAC ratio intervals were calculated using CONFIDENCE.T in Excel and were less then ± 0.03 from the average (Supplemental Table 1). Z-tests for two population proportions were calculated on the site https://www.socscistatistics.com/tests/ztest/default2.aspx (used on February 19, 2022). We used numbers with two significant figures after the decimal point, and we used normal rounding (0.284 was rounded to 0.28 and 0.285 was rounded to 0.29).

## 3. Results

A total of 20 sperm samples were included in the analysis. 10 samples were obtained from men with previously reported pregnancies (referred to as **fertile men**), and 10 samples were obtained from men that were part of a couple diagnosed with unexplained infertility (referred to as **unexplained infertility patients**). The two groups of men had similar average age and semen analysis parameters (Table 2). As sperm cells differentiate, they lose cytoplasm and become denser; this difference is used to select the higher quality sperm found in the pellet during differential centrifugation (Oshio et al., 1987; Sakkas, 2013). Therefore, every cryopreserved sample was processed using gradient centrifugation, which resulted in two fractions: a fraction with a pellet of dense sperm, regarded as higher quality and referred to as higher quality sperm (Fácio et al., 2016; Karamahmutoglu et al., 2014), and a second fraction with an interface that accumulates lighter sperm, regarded as lower quality and referred to as lower quality sperm (Branigan et al., 1999).

In total, fertility status and fraction type produced four sperm groups, each with hypothetically distinct quality levels (Fig. 2A, Supplemental Table 1): (i) fertile higher quality sperm (Fig. 2B); (ii) fertile lower quality sperm (Fig. 2B); (iii) higher quality sperm from patients with unexplained infertility (Fig. 2C); and (iv) lower quality sperm from patients with unexplained infertility (Fig. 2D). These four groups enabled us to characterize the differences in sperm centriole quality between individuals with and without unexplained infertility. Fertile higher quality sperm will be referred to as the *Reference Population* (Fig. 2A).

(A) Breakdown of the four populations used. (B-E) For each of the nine FRAC ratio variables, the reference range is indicated by a thick line with brackets. Thinner lines with a dot indicate the 95% confidence range and the mean of a sample FRAC ratio. Blue arrows indicate 95% confidence ranges that are up to 1 SD outside the reference range for each variable. Orange arrows indicate 95% confidence ranges that are more than 1 SD outside the reference range for each variable. (B) The 95% confidence ranges of fertile higher quality sperm (the reference population) relative to the reference range. (C) The 95% confidence ranges of fertile lower quality sperm relative to the reference range. (D) The 95% confidence ranges of higher quality sperm with unexplained infertility relative to the reference range. Three men had four values outside the reference ranges. (E) The 95% confidence ranges of lower quality sperm with unexplained infertility relative to the reference range. Four men had seven values outside the reference ranges. Inside each panel, the same color represents the same individual.

### 3.1. Higher quality sperm of fertile individuals had mean FRAC ratios with approximately Gaussian distribution and relatively small range

The reference population was comprised of fertile individuals with prior paternity and a normal semen analysis. We tested for normality and found that the five tests used (Shapiro-Wilk, D’Agostino-Pearson, Jarque-Bera, Cramer-von Mises, and Anderson-Darling) did not reject the hypothesis that the data set is normally distributed in the cases of acetylated tubulin, tubulin DC and Ax, and POC1B DC and Ax (Supplementary Data 2). The tests produced mixed conclusions as to whether the reference population was normally distributed for POC1B PC. The five tests found that the reference population was not normally distributed for tubulin PC. Additionally, skewness and kurtosis were normal (between −1 and +1 and between −3 and +3, respectively) for all nine variables, with the exception of PC tubulin skewness (Supplemental Table 2). This analysis indicates that most of the reference population variables had approximately Gaussian distribution.

The FRAC ratio has a minimum score of 0 and a maximum score of 1 (100%); the range between these minimum and maximum ratios defines the FRAC dynamic range. To study the variability in the reference population variables, we determined the *Population Range Difference*, which is defined as the range between these minimum and maximum mean FRAC ratios in the reference population for a specific biomarker at a specific location (Supplemental Table 3). The reference population range difference for the nine variables varied from 3% to 17%. Acetylated tubulin population range differences were 3%, 11%, and 11%; tubulin range differences were 10%, 7%, and 14%; and POC1B range differences were 12%, 16%, and 17% of the assay dynamic range in the PC, DC, and Ax, respectively. The range differences in fertile men are like those of infertile men with eumorphic sperm (tubulin: 8%, 9%, and 12%; POC1B: 14%, 21%, and 20%; P = 0.25 and 0.06 (Turner et al., 2021). Overall, the reference population range is small, allowing for the detection of small deviations and contributing to FRAC assay sensitivity.

To identify the individual mean FRAC ratio with a 95% probability of belonging to the reference population, we calculated the mean of the 10 individual mean FRAC ratios in the reference population ± two standard deviations (SDs), which we refer to as the *Reference Range* (Fig. 2B) (Turner et al., 2021). A mean FRAC ratio was considered an outlier when its 95% confidence interval was fully outside the reference range.

A sample was considered to have an *Optimal FRAC score* if the 95% confidence intervals of all nine mean FRAC ratios of that sample were fully or partially within the reference range. Samples with a mean FRAC ratio 95% confidence interval that fell outside the reference range for *any* of the nine variables were deemed to have a *Sub-Optimal FRAC score*.

To compare the four sperm populations, we counted the number of outlier values out of the 90 (each population was comprised of 10 individuals, each with nine mean FRAC ratios) measured values (Fig. 1E). The 95% confidence intervals of all 90 mean FRAC ratios were fully or partially within the reference range, and all 10 higher quality sperm samples were considered to have optimal FRAC scores (Fig. 2B).

### 3.2. Fertile lower quality sperm had normal centrioles

During sperm differentiation, centrioles are also being remodeled (Fishman et al., 2018). Therefore, we hypothesized that the less dense and thus less mature sperm found in the interface would have reduced centriolar quality. A past study of infertile men with eumorphic sperm found an almost significant increase in suboptimal centriole incidence using mean ratio values (4/9 versus 3/22, P = 0.06) (Turner et al., 2021). Here, using mean FRAC ratio 95% confidence intervals, we found that none of the 90 values of the 10 lower quality sperm samples from fertile individuals had suboptimal FRAC scores. Therefore, the FRAC scores of lower versus higher quality sperm from fertile men were not significantly different (0/90 versus 0/90, P = 1).

### 3.3. 30% of patients with unexplained infertility had suboptimal centrioles in the higher quality sperm fraction

Some reports suggest that patients with unexplained infertility have defective sperm centrioles (Garanina et al., 2019). Therefore, we hypothesized that FRAC could identify suboptimal sperm centrioles in some individuals with unexplained infertility. Hence, the objective of this analysis was to test if acetylated tubulin, tubulin, and POC1B, can identify centriolar anomalies in unexplained infertility samples.

We analyzed the mean ratios of higher quality sperm from 10 patients with unexplained infertility. We found that seven individuals (5, 9, 11, 13, 15, 17, and 20) had an optimal FRAC score with no outlier mean FRAC ratio 95% confidence intervals (Fig. 2D). In contrast, three patients had suboptimal FRAC scores. Patient 3 had a single outlier variable with a mean FRAC ratio 95% confidence interval that fell within up to 1 SD outside the reference range. Patient 6 had a single outlier variable with a mean FRAC ratio 95% confidence interval that fell more than 1 SD outside the reference range. Patient 16 had two mean FRAC ratios with 95% confidence intervals outside the reference range; one is within 1 SD outside the reference range, and one is more than 1 SD outside the reference range. In total, unexplained infertility higher quality sperm had four outlier values out of the 90 analyzed (4/90 versus 0/90 in the reference population, P = 0.043). These results suggest that the patients with unexplained infertility have more suboptimal centrioles than fertile men in their high-quality sperm fraction, a finding that is nearly statistically significant (3/10, versus 0/10, P = 0.06).

### 3.4. 40% of unexplained infertility patients had lower quality centrioles in the lower quality sperm fraction than those of fertile lower quality sperm individuals

We also tested if reduced centriolar quality could be detected when comparing lower quality sperm from men with unexplained infertility with lower quality sperm from fertile men. We found that four of the 10 lower quality sperm samples from patients with unexplained infertility (patients 3, 6, 11, and 16) had mean ratio 95% confidence intervals outside the reference range. Note that three of these four patients (patients 3, 6, and 16) also had abnormal FRAC scores in their higher quality sperm fraction, further supporting the conclusion that they had abnormal sperm. In total, unexplained infertility lower quality sperm had seven outlier values out of the 90 analyzed (7/90 versus 0/90 in the reference population, P = 0.007). This is also statistically significant compared to fertile men higher or lower quality sperm (0/10 versus 4/10, p = 0.025). This observation suggests that 40% of unexplained infertility patients had lower quality centrioles in the lower quality sperm fraction than those of fertile lower quality sperm. This finding suggests that FRAC analysis of lower quality sperm of unexplained infertility patients may be more likely to determine male causality for unexplained infertility in couples.

### 3.5. FRAC score identifies the male factor at the population and individual level

In this study, we analyzed 10 unexplained infertility patients in terms of three biomarkers at three locations in two fractions, resulting in 180 mean FRAC ratios for the fertile men population and 180 mean FRAC ratios for unexplained infertility patients (Fig. 1D). All 180 studied mean FRAC ratios in the fertile population were within the reference population. Eleven of the 180 studied mean FRAC ratios in the infertile patients were outliers (i.e., outside the 95% confidence range) (P = 0.0008). This analysis suggests that FRAC can identify a statistically significant difference between fertile and unexplained infertility populations.

At the level of individual men, FRAC found four unexplained infertility patients with outliers. The differences in the findings suggest that the unexplained infertility patients can be grouped into one of four categories:

**Normal male fertility:** Patients with no outliers in their higher and lower quality sperm. There were six unexplained infertility patients in our cohort with normal sperm centrioles – patients 5, 9, 13, 15, 17, and 20.

**Possible male factor:** Patients with one lower quality sperm outlier that is ≤ 1 SD away from the reference distribution – patient 11.

**Likely male factor:** Patients with one higher quality sperm outlier that is ≤ 1 SD away from the reference distribution and one or more lower quality sperm outliers – patient 3.

**Very likely male factor:** Patients with one or more higher quality sperm outliers that are > 1 SD away from the reference distribution and one or more lower quality sperm outliers that are > 1 SD away from the reference distribution – patients 6 and 16.

### 3.6. Acetylated tubulin is the most common biomarker in lower quality centrioles

We analyzed three centriolar biomarkers: two are structural proteins (tubulin and POC1B), and one is a post-translational modification (acetylated tubulin). We also examined which of these three biomarkers is most sensitive to change by counting the number of oudier mean FRAC ratios found in each of the four sperm populations (Table 3). We found that the biomarker that most commonly produces outliers is acetylated tubulin (twice the combined number of outliers in both tubulin and POC1B), suggesting that it is the best candidate for identifying reduction in centriolar quality. Accordingly, F-tests found that the PC acetylated tubulin standard deviations between fertile higher quality sperm and fertile lower quality sperm, infertile higher quality sperm, or infertile lower quality sperm were statistically different (P = 0.0052, 0.003, or 0.0000068, respectively) (Supplemental Table 4). Interestingly, total levels of acetylated α-tubulin detected by western blot are reduced in individuals with poor sperm motility (Bhagwat et al., 2014). Therefore, it is important to note that FRAC ratios represent variations in relative localizations and do not simply relate to expression levels.

### 3.7. The FRAC method has high rater reproducibility

All data presented in this study were quantified by one highly experienced rater. However, to gain insight into the reproducibility of the quantification step, we assessed rater performance in 10 individuals (five of the fertile males and five of the infertile males) (Supplemental Fig. 1). The selection of these samples was blinded. Because there is no “Gold Standard” for centriolar quality, we used the ICC, a widely used reliability index in test-retest, intra-reader, and inter-reader reliability analyses (Koo and Li, 2016). These additional raters were less experienced than the original rater; however, we found that they had excellent ICCs of greater than 0.90. Notably, most (16/20 =80%) of the ICCs were above 98%. Importantly, the two patients identified as having a male factor by our highly experienced rater were also found to have outliers by the additional raters. As a reference, the ICCs of standard semen parameters were reported to be moderate to high: semen volume, 0.70; sperm concentration, 0.89; sperm motility, 0.58; sperm morphology,60; total motile sperm count, 0.73 (Leushuis et al., 2010). These data demonstrate that FRAC is a promising assay with high reproducibility across raters.

## 4. Discussion

In this pilot study, the FRAC assay identified four out of 10 patients with unexplained infertility as having outlier FRAC values. This information can help patients with unexplained infertility and the clinicians treating them by informing them of (i) the infertile partner in the couple, i.e., the male if he receives an abnormal FRAC score; (ii) the gamete that would need to be replaced in the case of repetitive intracytoplasmic sperm injection (ICSI) treatment failure, i.e., the sperm if it receives an abnormal FRAC score; and (iii) a potential cause for infertility, i.e., the centriole. Altogether, the FRAC method can benefit infertility patients by reducing their uncertainties and cost and shortening the time to pregnancy, resulting in less stress on the couple’s relationship.

Identifying the male contribution to infertility is critical for improving infertility treatments and women’s health. A majority of physicians surveyed asserted that the development of a sperm test to characterize male factor infertility in cases of unexplained couple infertility would be helpful clinically and result in changes to practice patterns (Patel et al., 2019b). The consequences of failing to recognize the male contribution to infertility include reduced male fertility research and inadequate treatment of male fertility (Barratt et al., 2018; De Jonge and Barratt, 2019). This is evident in current clinical practice, with infertility treatments primarily using assisted reproductive technologies (ART), which are performed on the woman’s body, potentially impacting her health (Avidor-Reiss and Schon, 2021; Turner et al., 2020). An additional consequence is that women are often mislabeled as the cause of infertility, inducing psycho-social stress in front of their families and communities (Nasim et al., 2019; Webair et al., 2021).

A recent follow-up study to an NIH-supported Fast Track and Standard Treatment Trial (FASTT) involving 286 couples with unexplained infertility who were questioned via a telephone survey (56.9% replied) reported that out of the 194 couples that continued to try to conceive naturally, 101 achieved live birth (67.8%) (Vaughan et al., 2022). In addition, 94 women achieved a live birth via intrauterine insemination or in vitro fertilization (IVF). Therefore, it is important to determine if advanced sperm testing can help stratify patients with unexplained infertility into those that do not need further treatment, those that are likely to need treatment to conceive, and those that are unlikely to conceive even with currently available treatment.

For a couple with unexplained infertility, deciding which gamete to change when regular ICSI fails is complicated. There are no universally accepted guidelines for this type of situation, and clinicians must weigh the circumstances of each couple when approaching this decision. One way to guide the infertile couple is to employ more advanced testing, such as genetic testing (Sang et al., 2021). Complementary approaches that identify environmental effects can study sperm component composition, structure, and function. By its nature, FRAC is a method that provides information about sperm centriolar composition irrespective of genetic or environmental causes. Using multiple biomarkers, FRAC can provide information on centriolar structure by studying structural proteins, such as tubulin and POC1B, and centriole functional stats by studying regulated modification, such as tubulin acetylation.

POC1B is a structural protein that functions specifically in the centriole (Keller et al., 2009; Khire et al., 2016; Pearson et al., 2009; Roosing et al., 2014; Venoux et al., 2013). In this study, none of the 10 unexplained infertility patients had abnormal mean FRAC ratios with POC1B as a biomarker (i.e., normal sperm morphology among other standard semen features). In contrast, POC1B FRAC outliers were found in four of the nine infertile patients with abnormal sperm morphology studied by Turner et al. (2021). Interestingly, POC1B (aka WDR51B) mRNA is downregulated tenfold in sperm with abnormal morphology (i.e., teratozoospermia) (Platts et al., 2007). These two observations suggest that sperm with abnormal morphology are associated with anomalous POC1B protein localization and reduced POC1B mRNA and likely have a structural defect in the centriole.

Tubulin acetylation is a marker of stable microtubules (Guichard et al., 2021), and its high levels in the PC and DC of fertile men is consistent with their stable microtubules. One of the unexpected findings in this study is that of the biomarkers analyzed, acetylated tubulin shows the largest difference between fertile and unexplained infertility men. This may be because acetylated tubulin is a post-translational modification, while the other markers analyzed, tubulin and POC1B, are structural proteins. Acetylated tubulin is more differential, possibly because post-translational modification is more liable to change and can be added or removed quickly in response to signaling in the sperm cell. In mammals, the level of acetylated tubulin is governed mainly by the opposing actions of α-tubulin acetyltransferase 1 (ATAT1) and histone deacetylase 6 (HDAC6) (Li and Yang, 2015), with mRNAs for both being found in spermatozoa (Jodar et al., 2015). Acetylated tubulin levels in the cell are regulated. For example, Paxillin, a focal adhesion scaffold protein, interacts with HDAC6 and inhibits its deacetylase activity to upregulate microtubule acetylation during cell invasion and migration (Deakin and Turner, 2014). It would be important to understand the molecular pathways that regulate acetylated tubulin levels in the sperm cell.

The observation that acetylated tubulin is superior to structural protein as a biomarker may suggest that better, alternative markers may be other posttranslational modifications, such as phosphorylation, glutamination, and de-tyrosination. Investigating other posttranslational modifications is of particular interest, as the most common antibody against acetylated tubulin (6–11B-1) shows high levels of labeling in the acrosomal area in many sperm. This acrosomal labeling complicates the use of flow cytometry in evaluating sperm centrioles, an increasingly important tool for sperm analysis (DeJarnette et al., 2021). In this case, a method that is based on imaging a particular location, like FRAC, is advantageous.

The underlying mechanism leading to sperm centriole defects, and whether it is genetic or environment, is unknown. Yet, several centrosomal genes were implicated in infertility including CETN1, CEP131, Cep112, CEP128, CEP135, CEP63, POC1A, CEP78, DZIP1, WDR16, TSGA10, SPATC1L, and WDR62 (Ascari et al., 2020; Avasthi et al., 2013; Geister et al., 2015; Hall et al., 2013; Ho et al., 2021; Li et al., 2022; Lv et al., 2020; Marjanovic et al., 2015; Sha et al., 2017, 2018, 2020; Tapia Contreras and Hoyer-Fender, 2020; Zhang et al., 2022). The FRAC ratio is a method to normalize any biomarkers based on localization in the centriole. Once a genetic cause of centriole-based infertility is identified, FRAC can be used with antibodies that label the identified biomarker.

We found outlier FRAC scores in 40% of patients within unexplained infertility couples; this is a high rate considering that the centriole is only one of many sperm components that can contribute to unexplained infertility. This high rate raises the question: *why is FRAC so efficient in identifying a male factor in unexplained infertility couples?* There are two ways to theoretically explain this unexpected observation. First, owing to the lack of a convenient assay, it was not possible in the past to realize that centriolar changes are commonly associated with infertility. Secondly, FRAC score may be sensitive to sperm abnormalities and may provide information about causes of infertility that are not primarily centriolar. The latter explanation requires the centrioles to change in response to defects in other sperm structures, such as the nucleus, flagellum, or acrosome, either due to a pathological process or as an adaptation/compensation. It is interesting in this context that centrosomes are thought to act as a signal integration site (Arquint et al., 2014; Campbell et al., 2020; Doxsey et al., 2005; Joukov and De Nicolo, 2019; Shahi et al., 2022). If sperm centrioles act similarly, they may respond to signals initiated by pathological processes in the tail or head.

In its current form, the FRAC assay is performed in a research lab by scientists, a time-consuming and expensive procedure. In the future, this assay needs to be designed for the clinical environment to be used by trained technicians working in a typical andrology lab that routinely tests sperm samples provided by the patient for standard semen analysis. This design should include a simple FRAC staining kit, an automated fluorescent microscope, and FRAC software to quantify the sperm images and calculate the FRAC score.

This study is a small, retrospective, observational, epidemiologic pilot study, so caution should be exercised when interpreting the study findings. We studied a limited patient subset of 10 fertile men and 10 patients with unexplained infertility from one population in one location, Michigan. Thus, it may not be possible to generalize the findings to the general population or other sub-populations. However, our previous study included data from 33 other individuals from northeast Ohio (Turner et al., 2021) suggests that the conclusions may not be limited only to Michigan.

We found outlier FRAC scores in infertile men. These scores may represent a change in the centriole that is either a contributor to or a consequence of an underlying cause of infertility. These two possibilities can be distinguished in the future with a more detailed sperm analysis that includes other sperm factors as well as by using centrosome functional tests. The new WHO 6th edition (2022) guidelines include three types of semen analysis: basic, extended, and advanced (Boitrelle et al., 2021). The advanced category includes sperm DNA fragmentation and protein oxidation.

Our hypothesis is that sperm centriolar abnormalities cause male infertility, and FRAC is a sensitive, advanced sperm analysis tool that can identify male infertility in a broad spectrum of patients, including cases in which the centriole is not the primary cause. Future advanced sperm analysis may benefit from a multiparametric sperm analysis that also includes sperm centrioles. Future studies should further explore the following questions: (i) What is the prevalence of centriole-based infertility? Or, in other words, how often is centriolar abnormality the primary cause of idiopathic or unexplained infertility? (ii) Can the analysis of sperm DNA, RNA, proteins, or centrioles identify sperm deficiency in some unexplained infertility samples? Or, in other words, can multiparametric sperm analysis reproducibly identify male factors in unexplained infertile couples? (iii) Do the various sperm content tests identify male factor infertility in the same or distinct samples? Or, in other words, are the separate sperm component tests complementary or redundant?

## 5. Concluding remarks

Infertility treatment and, particularly, ART in the form of IVF or ICSI are physically, emotionally, and financially taxing (Kang et al., 2021). These adverse effects can potentially be minimized by advanced semen analysis, such as FRAC. FRAC is a promising assay based on centriolar staining that can identify a male factor in couples that, with current semen analysis, are diagnosed with unexplained infertility. FRAC can also identify a sperm centriolar anomaly in patients with male factor infertility. More research is needed to determine how FRAC can benefit patients with infertility.

## Supplementary Material

Supplementary information

## Figures and Tables

**Table 1:** Antibodies used in this study.

Target	Name, company name, catalog number, batch number	Dilution	Validation	Role
POC1B	Rabbit anti-POC1B – 10537	1:100	(Fishman et al., 2018; Turner et al., 2021)	Primary antibody
Acetylated tubulin	Mouse anti-acetylated tubulin	1:100	(Fishman et al., 2018	Primary antibody
Tubulin	Sheep anti-tubulin (Cytoskeleton, Inc.)	1:600	(Fishman et al., 2018; Turner et al., 2021)	Primary antibody
Donkey anti-rabbit Alexa 647	Donkey anti-Rabbit IgG (H+L) Highly Cross-Adsorbed Secondary Antibody, Alexa Fluor Plus 647, Thermo Fisher Scientific, A-32795	1:400	NA	Secondary antibody
Donkey anti-mouse Daylight 488	Mouse IgG (H+L) Cross-Adsorbed Secondary Antibody, Thermo Fisher Scientific, SA5–10166	1:400	NA	Secondary antibody
Donkey anti-sheep Alexa 555	Donkey anti-Sheep Alexa 555, Thermo Fisher Scientific, A-21436	1:1000	NA	Secondary antibody

NA – not applicable

**Table 2:** Semen analysis properties in the studied populations of fertile individuals and unexplained infertility patients are similar. Fertile individuals had prior paternity with normal semen analysis. T-tests for all six semen measurements are above 0.05, indicating that the fertile individuals and unexplained infertility patients have similar general characteristics.

	Number of individuals	Age (years)	Semen volume (mL)	Sperm concentration (x 10^6)	Motility (%)	Forward progression (%)	Morphology (%)
Fertile men	10	35.2	4.3	110	58	50	12
Unexplained infertility patients	10	36.2	4.2	123	65	56	13
P (T-test)		0.59	0.90	0.55	0.10	0.12	0.64

**Table 3:** Acetylated tubulin is the most common marker of lower quality centrioles: The table depicts the number of outlier mean FRAC ratios in the 20 individuals studied.

		Fertile		Unexplained infertility			
Marker	Location	Higher quality	Lower quality	Higher quality	Lower quality	Total	Overall
Ace Tubulin	PC	0	0	2	4	6	8
	DC	0	0	0	0	0	
	Ax	0	0	1	1	2	
Tubulin	PC	0	0	0	0	0	3
	DC	0	0	0	1	1	
	Ax	0	0	1	1	2	
POC1B	PC	0	0	0	0	0	0
	DC	0	0	0	0	0	
	Ax	0	0	0	0	0	

## Data Availability

Data will be made available on request.

## Abbreviations:

FRAC: Fluorescence-Based Ratiometric Analysis of Sperm Centrioles
PC: Proximal centriole
DC: Distal centriole
