# Supplementary material for: Sperm centriole assessment identifies male factor infertility in couples with unexplained infertility – a pilot study
Source: Eur J Cell Biol. Author manuscript; Available in PMC 2022 Nov 25. (PMC9692136; doi:10.1016/j.ejcb.2022.151243)
Supplement: Supplementary information [file NIHMS1848050-supplement-Supplementary_information.pdf]

Supplementary information

Supplemental Figure 1: the FRAC method shows high rater reproducibility.

We compared the performance of our highly experienced rater (red) with five additional raters of various experience levels (blue, magenta, orange, black, and green) in samples of 10 distinct individuals. Excellent intraclass correlation coefficients (ICC) were found in all 10 individuals. For each individual, the reference range (plotted using the data of the highly experienced rater, as shown in **Fig 2**) is indicated by a thick line with brackets. A dot with a line indicates the mean FRAC ratio and 95% confidence interval of one rater. A green square marks outlier values found in infertile patients. The ratio of the number of raters who found the values to be outliers is indicated.

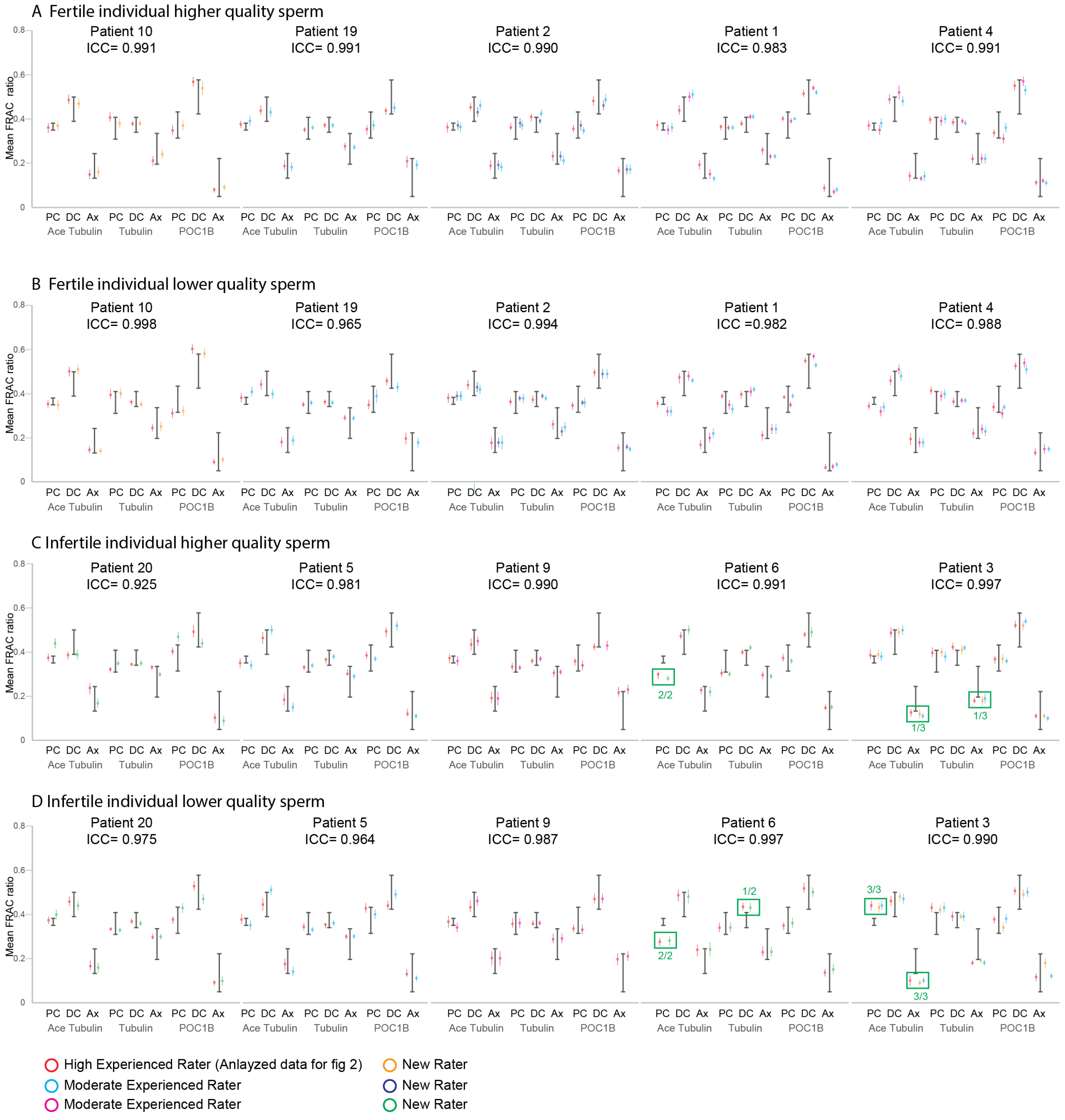

Supplemental Table 1: Mean FRAC values and 95% confidence intervals of the 20 individuals in this study.

Green highlights mark the reference range upper and lower values and ± 1 SD values outside the reference range. Orange highlights indicate 95% confidence intervals that are up to 1 SD outside the reference range for each variable. Blue highlights indicate 95% confidence intervals that are more than 1 SD outside the reference range for each variable.

Reference population

1 SD outside the refence range	0.39	0.53	0.27	0.43	0.43	0.37	0.46	0.62	0.25
Lower reference range	**0.38**	**0.50**	**0.24**	**0.41**	**0.41**	**0.34**	**0.43**	**0.58**	**0.21**
Uper reference range	**0.35**	**0.39**	**0.13**	**0.31**	**0.34**	**0.20**	**0.31**	**0.42**	**0.04**
1 SD outside the refence range	0.34	0.36	0.10	0.29	0.32	0.16	0.28	0.39	0.00

Patient 10 higher quality sperm

	Ace Tub PC	Ace Tub DC	Ace Tub Ax	Tub PC	Tub DC	Tub Ax	POC1B PC	POC1B DC	POC1B Ax
Mean	0.36	0.49	0.15	0.41	0.38	0.21	0.35	0.57	0.08
SD	0.11	0.11	0.11	0.11	0.07	0.10	0.10	0.12	0.07
N	104	104	104	104	104	104	104	104	104
Significance l	0.05	0.05	0.05	0.05	0.05	0.05	0.05	0.05	0.05
CI	0.34 - 0.38	0.47 - 0.51	0.13 - 0.17	0.39 - 0.43	0.37 - 0.39	0.19 - 0.23	0.33 - 0.37	0.55 - 0.59	0.07 - 0.09
Range	0.04	0.04	0.04	0.04	0.03	0.04	0.04	0.05	0.03

Patient 10 lower quality sperm

	Ace Tub PC	Ace Tub DC	Ace Tub Ax	Tub PC	Tub DC	Tub Ax	POC1B PC	POC1B DC	POC1B Ax
Mean	0.35	0.50	0.14	0.40	0.36	0.24	0.31	0.60	0.09
SD	0.10	0.10	0.09	0.11	0.06	0.08	0.09	0.10	0.06
N	121	121	121	121	121	121	121	121	121
Significance l	0.05	0.05	0.05	0.05	0.05	0.05	0.05	0.05	0.05
CI	0.34 - 0.37	0.48 - 0.52	0.13 - 0.16	0.38 - 0.42	0.35 - 0.37	0.23 - 0.26	0.29 - 0.33	0.58 - 0.62	0.08 - 0.1
Range	0.04	0.04	0.03	0.04	0.02	0.03	0.03	0.04	0.02

Patient 19 higher quality sperm

	Ace Tub PC	Ace Tub DC	Ace Tub Ax	Tub PC	Tub DC	Tub Ax	POC1B PC	POC1B DC	POC1B Ax
Mean	0.38	0.44	0.19	0.35	0.37	0.28	0.35	0.44	0.21
SD	0.09	0.09	0.10	0.06	0.05	0.07	0.10	0.09	0.13
N	106	106	106	106	106	106	106	106	106
Significance l	0.05	0.05	0.05	0.05	0.05	0.05	0.05	0.05	0.05
CI	0.36 - 0.39	0.42 - 0.46	0.17 - 0.21	0.34 - 0.36	0.36 - 0.38	0.26 - 0.29	0.33 - 0.37	0.42 - 0.45	0.18 - 0.23
Range	0.03	0.04	0.04	0.02	0.02	0.03	0.04	0.03	0.05

Patient 19 lower quality sperm

	Ace Tub PC	Ace Tub DC	Ace Tub Ax	Tub PC	Tub DC	Tub Ax	POC1B PC	POC1B DC	POC1B Ax
Mean	0.38	0.44	0.18	0.35	0.36	0.29	0.35	0.46	0.20
SD	0.10	0.10	0.10	0.05	0.06	0.07	0.12	0.10	0.14
N	110	110	110	110	110	110	110	110	110
Significance l	0.05	0.05	0.05	0.05	0.05	0.05	0.05	0.05	0.05
CI	0.36 - 0.4	0.42 - 0.46	0.16 - 0.2	0.34 - 0.36	0.35 - 0.37	0.28 - 0.3	0.33 - 0.37	0.44 - 0.47	0.17 - 0.22
Range	0.04	0.04	0.04	0.02	0.02	0.02	0.04	0.04	0.05

Patient 2 higher quality sperm

	Ace Tub PC	Ace Tub DC	Ace Tub Ax	Tub PC	Tub DC	Tub Ax	POC1B PC	POC1B DC	POC1B Ax
Mean	0.36	0.45	0.19	0.36	0.41	0.23	0.35	0.48	0.16
SD	0.10	0.09	0.11	0.09	0.08	0.10	0.09	0.11	0.09
N	117	117	117	117	117	117	117	117	117
Significance l	0.05	0.05	0.05	0.05	0.05	0.05	0.05	0.05	0.05
CI	0.34 - 0.38	0.44 - 0.47	0.17 - 0.21	0.35 - 0.38	0.39 - 0.42	0.21 - 0.25	0.34 - 0.37	0.46 - 0.5	0.15 - 0.18
Range	0.04	0.03	0.04	0.03	0.03	0.04	0.03	0.04	0.03

Patient 2 lower quality sperm

	Ace Tub PC	Ace Tub DC	Ace Tub Ax	Tub PC	Tub DC	Tub Ax	POC1B PC	POC1B DC	POC1B Ax
Mean	0.38	0.44	0.18	0.36	0.37	0.26	0.35	0.50	0.16
SD	0.11	0.09	0.13	0.08	0.07	0.10	0.07	0.08	0.07
N	97	97	97	97	97	97	97	97	97
Significance l	0.05	0.05	0.05	0.05	0.05	0.05	0.05	0.05	0.05
CI	0.36 - 0.4	0.42 - 0.46	0.15 - 0.2	0.35 - 0.38	0.36 - 0.39	0.24 - 0.28	0.33 - 0.36	0.48 - 0.51	0.14 - 0.17
Range	0.04	0.04	0.05	0.03	0.03	0.04	0.03	0.03	0.03

Patient 8 higher quality sperm

	Ace Tub PC	Ace Tub DC	Ace Tub Ax	Tub PC	Tub DC	Tub Ax	POC1B PC	POC1B DC	POC1B Ax
Mean	0.36	0.44	0.20	0.33	0.36	0.31	0.39	0.46	0.15
SD	0.12	0.12	0.13	0.06	0.06	0.07	0.08	0.10	0.08
N	102	102	102	102	102	102	102	102	102
Significance l	0.05	0.05	0.05	0.05	0.05	0.05	0.05	0.05	0.05
CI	0.34 - 0.39	0.42 - 0.46	0.17 - 0.22	0.32 - 0.34	0.35 - 0.37	0.3 - 0.33	0.37 - 0.4	0.44 - 0.48	0.14 - 0.16
Range	0.05	0.05	0.05	0.02	0.02	0.03	0.03	0.04	0.03

Patient 8 lower quality sperm

	Ace Tub PC	Ace Tub DC	Ace Tub Ax	Tub PC	Tub DC	Tub Ax	POC1B PC	POC1B DC	POC1B Ax
Mean	0.36	0.44	0.20	0.34	0.36	0.30	0.40	0.47	0.13
SD	0.11	0.12	0.13	0.06	0.05	0.06	0.11	0.12	0.08
N	109	109	109	109	109	109	109	109	109
Significance l	0.05	0.05	0.05	0.05	0.05	0.05	0.05	0.05	0.05
CI	0.34 - 0.38	0.42 - 0.46	0.17 - 0.22	0.33 - 0.35	0.35 - 0.36	0.29 - 0.31	0.38 - 0.42	0.45 - 0.49	0.12 - 0.15
Range	0.04	0.05	0.05	0.02	0.02	0.02	0.04	0.04	0.03

Patient 1 higher quality sperm

	Ace Tub PC	Ace Tub DC	Ace Tub Ax	Tub PC	Tub DC	Tub Ax	POC1B PC	POC1B DC	POC1B Ax
Mean	0.37	0.44	0.19	0.36	0.38	0.26	0.40	0.51	0.09
SD	0.09	0.12	0.13	0.06	0.05	0.08	0.05	0.09	0.06
N	109	109	109	109	109	109	109	109	109
Significance l	0.05	0.05	0.05	0.05	0.05	0.05	0.05	0.05	0.05
CI	0.35 - 0.39	0.42 - 0.46	0.17 - 0.21	0.35 - 0.37	0.37 - 0.39	0.24 - 0.27	0.39 - 0.41	0.5 - 0.53	0.07 - 0.1
Range	0.03	0.04	0.05	0.02	0.02	0.03	0.02	0.04	0.02

Patient 1 lower quality sperm

	Ace Tub PC	Ace Tub DC	Ace Tub Ax	Tub PC	Tub DC	Tub Ax	POC1B PC	POC1B DC	POC1B Ax
Mean	0.36	0.47	0.17	0.39	0.40	0.21	0.39	0.55	0.07
SD	0.09	0.11	0.11	0.07	0.09	0.10	0.05	0.07	0.05
N	111	111	111	111	111	111	111	111	111
Significance l	0.05	0.05	0.05	0.05	0.05	0.05	0.05	0.05	0.05
CI	0.34 - 0.37	0.45 - 0.49	0.15 - 0.19	0.38 - 0.4	0.38 - 0.41	0.19 - 0.23	0.38 - 0.39	0.54 - 0.56	0.06 - 0.08
Range	0.03	0.04	0.04	0.03	0.03	0.04	0.02	0.03	0.02

Patient 14 higher quality sperm

	Ace Tub PC	Ace Tub DC	Ace Tub Ax	Tub PC	Tub DC	Tub Ax	POC1B PC	POC1B DC	POC1B Ax
Mean	0.35	0.41	0.23	0.35	0.35	0.30	0.42	0.50	0.08
SD	0.09	0.08	0.11	0.07	0.05	0.08	0.10	0.08	0.07
N	106	106	106	106	106	106	106	106	106
Significance l	0.05	0.05	0.05	0.05	0.05	0.05	0.05	0.05	0.05
CI	0.34 - 0.37	0.4 - 0.43	0.21 - 0.25	0.34 - 0.36	0.34 - 0.36	0.28 - 0.31	0.4 - 0.44	0.48 - 0.52	0.07 - 0.09
Range	0.03	0.03	0.04	0.03	0.02	0.03	0.04	0.03	0.02

Patient 14 lower quality sperm

	Ace Tub PC	Ace Tub DC	Ace Tub Ax	Tub PC	Tub DC	Tub Ax	POC1B PC	POC1B DC	POC1B Ax
Mean	0.36	0.40	0.24	0.39	0.36	0.25	0.39	0.49	0.12
SD	0.10	0.08	0.12	0.10	0.07	0.08	0.09	0.10	0.07
N	114	114	114	114	114	114	114	114	114
Significance l	0.05	0.05	0.05	0.05	0.05	0.05	0.05	0.05	0.05
CI	0.34 - 0.38	0.39 - 0.42	0.22 - 0.26	0.38 - 0.41	0.34 - 0.37	0.24 - 0.27	0.38 - 0.41	0.47 - 0.51	0.11 - 0.13
Range	0.04	0.03	0.04	0.04	0.03	0.03	0.03	0.04	0.03

Patient 7 higher quality sperm

	Ace Tub PC	Ace Tub DC	Ace Tub Ax	Tub PC	Tub DC	Tub Ax	POC1B PC	POC1B DC	POC1B Ax
Mean	0.36	0.46	0.18	0.34	0.39	0.27	0.34	0.50	0.16
SD	0.09	0.09	0.09	0.07	0.07	0.08	0.07	0.08	0.07
N	101	101	101	101	101	101	101	101	101
Significance l	0.05	0.05	0.05	0.05	0.05	0.05	0.05	0.05	0.05
CI	0.34 - 0.38	0.45 - 0.48	0.16 - 0.19	0.32 - 0.35	0.38 - 0.4	0.26 - 0.29	0.33 - 0.35	0.49 - 0.52	0.14 - 0.17
Range	0.04	0.04	0.04	0.03	0.03	0.03	0.03	0.03	0.03

Patient 7 lower quality sperm

	Ace Tub PC	Ace Tub DC	Ace Tub Ax	Tub PC	Tub DC	Tub Ax	POC1B PC	POC1B DC	POC1B Ax
Mean	0.35	0.44	0.21	0.38	0.38	0.23	0.36	0.49	0.16
SD	0.13	0.13	0.15	0.12	0.09	0.10	0.12	0.12	0.08
N	101	101	101	101	101	101	101	101	101
Significance l	0.05	0.05	0.05	0.05	0.05	0.05	0.05	0.05	0.05
CI	0.33 - 0.38	0.41 - 0.47	0.18 - 0.24	0.36 - 0.41	0.37 - 0.4	0.21 - 0.25	0.33 - 0.38	0.46 - 0.51	0.14 - 0.17
Range	0.05	0.05	0.06	0.05	0.03	0.04	0.05	0.05	0.03

Patient 18 higher quality sperm

	Ace Tub PC	Ace Tub DC	Ace Tub Ax	Tub PC	Tub DC	Tub Ax	POC1B PC	POC1B DC	POC1B Ax
Mean	0.38	0.42	0.20	0.35	0.36	0.29	0.39	0.49	0.12
SD	0.09	0.09	0.10	0.10	0.05	0.08	0.10	0.11	0.07
N	103	103	103	103	103	103	103	103	103
Significance l	0.05	0.05	0.05	0.05	0.05	0.05	0.05	0.05	0.05
CI	0.36 - 0.4	0.4 - 0.43	0.18 - 0.22	0.33 - 0.37	0.35 - 0.37	0.27 - 0.3	0.37 - 0.41	0.47 - 0.51	0.11 - 0.14
Range	0.04	0.04	0.04	0.04	0.02	0.03	0.04	0.04	0.03

Patient 18 lower quality sperm

	Ace Tub PC	Ace Tub DC	Ace Tub Ax	Tub PC	Tub DC	Tub Ax	POC1B PC	POC1B DC	POC1B Ax
Mean	0.40	0.42	0.18	0.33	0.35	0.32	0.37	0.51	0.12
SD	0.11	0.10	0.11	0.05	0.05	0.06	0.10	0.11	0.07
N	103	103	103	103	103	103	103	103	103
Significance l	0.05	0.05	0.05	0.05	0.05	0.05	0.05	0.05	0.05
CI	0.38 - 0.42	0.4 - 0.43	0.16 - 0.2	0.32 - 0.34	0.34 - 0.36	0.31 - 0.33	0.35 - 0.39	0.49 - 0.53	0.11 - 0.13
Range	0.04	0.04	0.04	0.02	0.02	0.02	0.04	0.04	0.03

Patient 4 higher quality sperm

	Ace Tub PC	Ace Tub DC	Ace Tub Ax	Tub PC	Tub DC	Tub Ax	POC1B PC	POC1B DC	POC1B Ax
Mean	0.37	0.49	0.14	0.40	0.38	0.22	0.34	0.55	0.11
SD	0.11	0.10	0.10	0.09	0.07	0.09	0.07	0.09	0.06
N	111	111	111	111	111	111	111	111	111
Significance l	0.05	0.05	0.05	0.05	0.05	0.05	0.05	0.05	0.05
CI	0.35 - 0.39	0.47 - 0.51	0.12 - 0.16	0.38 - 0.41	0.37 - 0.4	0.2 - 0.24	0.32 - 0.35	0.53 - 0.57	0.1 - 0.12
Range	0.04	0.04	0.04	0.03	0.03	0.03	0.03	0.03	0.02

Patient 4 lower quality sperm

	Ace Tub PC	Ace Tub DC	Ace Tub Ax	Tub PC	Tub DC	Tub Ax	POC1B PC	POC1B DC	POC1B Ax
Mean	0.34	0.46	0.20	0.41	0.36	0.22	0.34	0.53	0.13
SD	0.10	0.11	0.11	0.09	0.07	0.09	0.08	0.10	0.06
N	101	101	101	101	101	101	101	101	101
Significance l	0.05	0.05	0.05	0.05	0.05	0.05	0.05	0.05	0.05
CI	0.33 - 0.36	0.44 - 0.48	0.17 - 0.22	0.4 - 0.43	0.35 - 0.38	0.2 - 0.24	0.32 - 0.36	0.51 - 0.54	0.12 - 0.15
Range	0.04	0.04	0.04	0.04	0.03	0.03	0.03	0.04	0.03

Patient 12 higher quality sperm

	Ace Tub PC	Ace Tub DC	Ace Tub Ax	Tub PC	Tub DC	Tub Ax	POC1B PC	POC1B DC	POC1B Ax
Mean	0.37	0.41	0.22	0.35	0.36	0.29	0.40	0.51	0.10
SD	0.10	0.10	0.10	0.09	0.06	0.09	0.11	0.11	0.08
N	111	111	111	111	111	111	111	111	111
Significance l	0.05	0.05	0.05	0.05	0.05	0.05	0.05	0.05	0.05
CI	0.35 - 0.39	0.4 - 0.43	0.2 - 0.24	0.33 - 0.36	0.35 - 0.37	0.28 - 0.31	0.38 - 0.42	0.48 - 0.53	0.08 - 0.11
Range	0.04	0.04	0.04	0.03	0.02	0.03	0.04	0.04	0.03

Patient 12 lower quality sperm

	Ace Tub PC	Ace Tub DC	Ace Tub Ax	Tub PC	Tub DC	Tub Ax	POC1B PC	POC1B DC	POC1B Ax
Mean	0.33	0.47	0.20	0.32	0.37	0.32	0.36	0.53	0.11
SD	0.08	0.10	0.13	0.05	0.06	0.07	0.08	0.08	0.07
N	97	97	97	97	97	97	97	97	97
Significance l	0.05	0.05	0.05	0.05	0.05	0.05	0.05	0.05	0.05
CI	0.31 - 0.34	0.45 - 0.49	0.18 - 0.23	0.31 - 0.33	0.36 - 0.38	0.3 - 0.33	0.35 - 0.38	0.51 - 0.55	0.09 - 0.12
Range	0.03	0.04	0.05	0.02	0.02	0.03	0.03	0.03	0.03

Unexplained infertility

Patient 20 higher quality sperm

	Ace Tub PC	Ace Tub DC	Ace Tub Ax	Tub PC	Tub DC	Tub Ax	POC1B PC	POC1B DC	POC1B Ax
Mean	0.37	0.39	0.24	0.32	0.34	0.33	0.40	0.49	0.10
SD	0.10	0.09	0.12	0.05	0.04	0.06	0.09	0.12	0.10
N	111	111	111	111	111	111	111	111	111
Significance l	0.05	0.05	0.05	0.05	0.05	0.05	0.05	0.05	0.05
CI	0.36 - 0.39	0.37 - 0.4	0.21 - 0.26	0.31 - 0.33	0.34 - 0.35	0.32 - 0.34	0.39 - 0.42	0.47 - 0.52	0.08 - 0.12
Range	0.04	0.03	0.05	0.02	0.02	0.02	0.03	0.05	0.04

Patient 20 lower quality sperm

	Ace Tub PC	Ace Tub DC	Ace Tub Ax	Tub PC	Tub DC	Tub Ax	POC1B PC	POC1B DC	POC1B Ax
Mean	0.37	0.46	0.17	0.33	0.37	0.30	0.38	0.53	0.09
SD	0.10	0.11	0.11	0.05	0.05	0.06	0.08	0.10	0.06
N	112	112	112	112	112	112	112	112	112
Significance l	0.05	0.05	0.05	0.05	0.05	0.05	0.05	0.05	0.05
CI	0.36 - 0.39	0.44 - 0.48	0.15 - 0.19	0.33 - 0.34	0.36 - 0.38	0.29 - 0.31	0.36 - 0.39	0.51 - 0.55	0.08 - 0.1
Range	0.04	0.04	0.04	0.02	0.02	0.02	0.03	0.04	0.02

Patient 5 higher quality sperm

	Ace Tub PC	Ace Tub DC	Ace Tub Ax	Tub PC	Tub DC	Tub Ax	POC1B PC	POC1B DC	POC1B Ax
Mean	0.35	0.46	0.19	0.33	0.36	0.31	0.39	0.49	0.12
SD	0.10	0.13	0.14	0.04	0.04	0.05	0.08	0.10	0.08
N	103	103	103	103	103	103	103	103	103
Significance l	0.05	0.05	0.05	0.05	0.05	0.05	0.05	0.05	0.05
CI	0.33 - 0.37	0.44 - 0.49	0.16 - 0.21	0.32 - 0.34	0.35 - 0.37	0.3 - 0.32	0.37 - 0.4	0.47 - 0.51	0.11 - 0.14
Range	0.04	0.05	0.05	0.02	0.02	0.02	0.03	0.04	0.03

Patient 5 lower quality sperm

	Ace Tub PC	Ace Tub DC	Ace Tub Ax	Tub PC	Tub DC	Tub Ax	POC1B PC	POC1B DC	POC1B Ax
Mean	0.38	0.45	0.18	0.34	0.35	0.30	0.43	0.44	0.13
SD	0.11	0.14	0.14	0.06	0.05	0.07	0.11	0.11	0.08
N	115	115	115	115	115	115	115	115	115
Significance l	0.05	0.05	0.05	0.05	0.05	0.05	0.05	0.05	0.05
CI	0.36 - 0.4	0.42 - 0.47	0.15 - 0.2	0.33 - 0.36	0.34 - 0.36	0.29 - 0.31	0.41 - 0.45	0.42 - 0.46	0.12 - 0.15
Range	0.04	0.05	0.05	0.02	0.02	0.03	0.04	0.04	0.03

Patient 13 higher quality sperm

	Ace Tub PC	Ace Tub DC	Ace Tub Ax	Tub PC	Tub DC	Tub Ax	POC1B PC	POC1B DC	POC1B Ax
Mean	0.38	0.40	0.22	0.37	0.36	0.27	0.39	0.47	0.13
SD	0.12	0.11	0.16	0.09	0.06	0.10	0.13	0.16	0.14
N	105	105	105	105	105	105	105	105	105
Significance l	0.05	0.05	0.05	0.05	0.05	0.05	0.05	0.05	0.05
CI	0.36 - 0.4	0.38 - 0.42	0.19 - 0.25	0.36 - 0.39	0.34 - 0.37	0.25 - 0.29	0.36 - 0.41	0.44 - 0.5	0.1 - 0.15
Range	0.04	0.04	0.06	0.03	0.02	0.04	0.05	0.06	0.05

Patient 13 lower quality sperm

	Ace Tub PC	Ace Tub DC	Ace Tub Ax	Tub PC	Tub DC	Tub Ax	POC1B PC	POC1B DC	POC1B Ax
Mean	0.40	0.43	0.17	0.40	0.37	0.23	0.40	0.51	0.09
SD	0.08	0.07	0.08	0.09	0.07	0.10	0.07	0.08	0.06
N	99	99	99	99	99	99	99	99	99
Significance l	0.05	0.05	0.05	0.05	0.05	0.05	0.05	0.05	0.05
CI	0.38 - 0.41	0.42 - 0.45	0.15 - 0.19	0.38 - 0.42	0.36 - 0.39	0.21 - 0.25	0.39 - 0.41	0.5 - 0.53	0.07 - 0.1
Range	0.03	0.03	0.03	0.04	0.03	0.04	0.03	0.03	0.02

Patient 9 higher quality sperm

	Ace Tub PC	Ace Tub DC	Ace Tub Ax	Tub PC	Tub DC	Tub Ax	POC1B PC	POC1B DC	POC1B Ax
Mean	0.37	0.43	0.19	0.33	0.36	0.31	0.36	0.42	0.22
SD	0.11	0.12	0.13	0.07	0.05	0.06	0.08	0.09	0.10
N	107	107	107	107	107	107	107	107	107
Significance l	0.05	0.05	0.05	0.05	0.05	0.05	0.05	0.05	0.05
CI	0.35 - 0.39	0.41 - 0.46	0.17 - 0.22	0.32 - 0.35	0.35 - 0.37	0.29 - 0.32	0.34 - 0.37	0.41 - 0.44	0.20 - 0.24
Range	0.04	0.04	0.05	0.03	0.02	0.02	0.03	0.03	0.04

Patient 9 lower quality sperm

	Ace Tub PC	Ace Tub DC	Ace Tub Ax	Tub PC	Tub DC	Tub Ax	POC1B PC	POC1B DC	POC1B Ax
Mean	0.37	0.43	0.20	0.36	0.36	0.29	0.34	0.47	0.20
SD	0.12	0.13	0.14	0.10	0.05	0.10	0.09	0.11	0.11
N	101	101	101	101	101	101	101	101	101
Significance l	0.05	0.05	0.05	0.05	0.05	0.05	0.05	0.05	0.05
CI	0.34 - 0.39	0.41 - 0.46	0.17 - 0.23	0.34 - 0.38	0.35 - 0.37	0.27 - 0.31	0.32 - 0.35	0.45 - 0.49	0.17 - 0.22
Range	0.05	0.05	0.06	0.04	0.02	0.04	0.04	0.04	0.04

Patient 17 higher quality sperm

	Ace Tub PC	Ace Tub DC	Ace Tub Ax	Tub PC	Tub DC	Tub Ax	POC1B PC	POC1B DC	POC1B Ax
Mean	0.33	0.43	0.24	0.33	0.36	0.31	0.39	0.46	0.16
SD	0.11	0.12	0.13	0.05	0.04	0.06	0.10	0.12	0.11
N	110	110	110	110	110	110	110	110	110
Significance l	0.05	0.05	0.05	0.05	0.05	0.05	0.05	0.05	0.05
CI	0.30 - 0.35	0.41 - 0.46	0.22 - 0.27	0.32 - 0.34	0.35 - 0.36	0.3 - 0.33	0.37 - 0.4	0.44 - 0.48	0.14 - 0.18
Range	0.04	0.05	0.05	0.02	0.02	0.02	0.04	0.04	0.04

Patient 17 lower quality sperm

	Ace Tub PC	Ace Tub DC	Ace Tub Ax	Tub PC	Tub DC	Tub Ax	POC1B PC	POC1B DC	POC1B Ax
Mean	0.33	0.45	0.22	0.33	0.36	0.31	0.38	0.50	0.12
SD	0.10	0.09	0.12	0.05	0.04	0.05	0.08	0.09	0.09
N	110	110	110	110	110	110	110	110	110
Significance l	0.05	0.05	0.05	0.05	0.05	0.05	0.05	0.05	0.05
CI	0.32 - 0.35	0.43 - 0.47	0.19 - 0.24	0.33 - 0.34	0.35 - 0.37	0.3 - 0.32	0.36 - 0.39	0.49 - 0.52	0.1 - 0.13
Range	0.04	0.04	0.05	0.02	0.02	0.02	0.03	0.03	0.03

Patient 11 higher quality sperm

	Ace Tub PC	Ace Tub DC	Ace Tub Ax	Tub PC	Tub DC	Tub Ax	POC1B PC	POC1B DC	POC1B Ax
Mean	0.33	0.45	0.22	0.32	0.38	0.30	0.38	0.47	0.15
SD	0.09	0.12	0.10	0.05	0.07	0.08	0.09	0.12	0.09
N	103	103	103	103	103	103	103	103	103
Significance l	0.05	0.05	0.05	0.05	0.05	0.05	0.05	0.05	0.05
CI	0.31 - 0.35	0.42 - 0.47	0.21 - 0.24	0.31 - 0.34	0.36 - 0.39	0.28 - 0.31	0.36 - 0.4	0.45 - 0.49	0.13 - 0.17
Range	0.03	0.04	0.04	0.02	0.03	0.03	0.04	0.05	0.03

Patient 11 lower quality sperm (Outlier infertile patient)

	Ace Tub PC	Ace Tub DC	Ace Tub Ax	Tub PC	Tub DC	Tub Ax	POC1B PC	POC1B DC	POC1B Ax
Mean	0.32	0.48	0.21	0.33	0.36	0.31	0.36	0.53	0.11
SD	0.10	0.11	0.10	0.04	0.05	0.05	0.11	0.12	0.08
N	104	104	104	104	104	104	104	104	104
Significance l	0.05	0.05	0.05	0.05	0.05	0.05	0.05	0.05	0.05
CI	0.30 - 0.34	0.45 - 0.5	0.19 - 0.23	0.32 - 0.34	0.35 - 0.37	0.3 - 0.32	0.34 - 0.38	0.5 - 0.55	0.1 - 0.13
Range	0.04	0.04	0.04	0.02	0.02	0.02	0.04	0.05	0.03

Patient 6 higher quality sperm (Outlier infertile patient)

	Ace Tub PC	Ace Tub DC	Ace Tub Ax	Tub PC	Tub DC	Tub Ax	POC1B PC	POC1B DC	POC1B Ax
Mean	0.30	0.47	0.23	0.30	0.40	0.30	0.37	0.48	0.15
SD	0.08	0.09	0.08	0.06	0.07	0.07	0.06	0.07	0.06
N	110	110	110	110	110	110	110	110	110
Significance l	0.05	0.05	0.05	0.05	0.05	0.05	0.05	0.05	0.05
CI	0.28 - 0.31	0.46 - 0.49	0.21 - 0.24	0.29 - 0.32	0.39 - 0.41	0.28 - 0.31	0.36 - 0.39	0.47 - 0.49	0.14 - 0.16
Range	0.03	0.03	0.03	0.02	0.03	0.03	0.02	0.03	0.02

Patient 6 lower quality sperm (Outlier infertile patient)

	Ace Tub PC	Ace Tub DC	Ace Tub Ax	Tub PC	Tub DC	Tub Ax	POC1B PC	POC1B DC	POC1B Ax
Mean	0.28	0.49	0.24	0.34	0.43	0.23	0.35	0.52	0.14
SD	0.08	0.12	0.12	0.08	0.07	0.09	0.07	0.10	0.08
N	96	96	96	96	96	96	96	96	96
Significance l	0.05	0.05	0.05	0.05	0.05	0.05	0.05	0.05	0.05
CI	0.26 - 0.29	0.46 - 0.51	0.21 - 0.26	0.32 - 0.36	0.42 - 0.45	0.21 - 0.25	0.33 - 0.36	0.5 - 0.54	0.12 - 0.15
Range	0.03	0.05	0.05	0.03	0.03	0.04	0.03	0.04	0.03

Patient 15 higher quality sperm

	Ace Tub PC	Ace Tub DC	Ace Tub Ax	Tub PC	Tub DC	Tub Ax	POC1B PC	POC1B DC	POC1B Ax
Mean	0.34	0.41	0.26	0.36	0.37	0.26	0.39	0.47	0.14
SD	0.10	0.10	0.15	0.09	0.07	0.12	0.10	0.10	0.13
N	104	104	104	104	104	104	104	104	104
Significance l	0.05	0.05	0.05	0.05	0.05	0.05	0.05	0.05	0.05
CI	0.32 - 0.36	0.39 - 0.43	0.23 - 0.28	0.35 - 0.38	0.36 - 0.39	0.24 - 0.29	0.37 - 0.41	0.45 - 0.49	0.11 - 0.16
Range	0.04	0.04	0.06	0.03	0.03	0.05	0.04	0.04	0.05

Patient 15 lower quality sperm

	Ace Tub PC	Ace Tub DC	Ace Tub Ax	Tub PC	Tub DC	Tub Ax	POC1B PC	POC1B DC	POC1B Ax
Mean	0.34	0.43	0.22	0.38	0.36	0.27	0.39	0.49	0.12
SD	0.10	0.09	0.11	0.08	0.05	0.08	0.10	0.10	0.08
N	109	109	109	109	109	109	109	109	109
Significance l	0.05	0.05	0.05	0.05	0.05	0.05	0.05	0.05	0.05
CI	0.33 - 0.36	0.42 - 0.45	0.20 - 0.24	0.36 - 0.39	0.35 - 0.36	0.25 - 0.28	0.37 - 0.41	0.47 - 0.51	0.1 - 0.13
Range	0.04	0.03	0.04	0.03	0.02	0.03	0.04	0.04	0.03

Patient 16 higher quality sperm (Outlier infertile patient)

	Ace Tub PC	Ace Tub DC	Ace Tub Ax	Tub PC	Tub DC	Tub Ax	POC1B PC	POC1B DC	POC1B Ax
Mean	0.30	0.41	0.29	0.32	0.37	0.31	0.36	0.46	0.17
SD	0.10	0.11	0.14	0.06	0.06	0.08	0.11	0.14	0.14
N	104	104	104	104	104	104	104	104	104
Significance l	0.05	0.05	0.05	0.05	0.05	0.05	0.05	0.05	0.05
CI	0.28 - 0.32	0.39 - 0.43	0.26 - 0.32	0.31 - 0.34	0.36 - 0.38	0.29 - 0.32	0.34 - 0.38	0.44 - 0.49	0.14 - 0.2
Range	0.04	0.04	0.06	0.02	0.02	0.03	0.04	0.05	0.05

Patient 16 lower quality sperm (Outlier infertile patient)

	Ace Tub PC	Ace Tub DC	Ace Tub Ax	Tub PC	Tub DC	Tub Ax	POC1B PC	POC1B DC	POC1B Ax
Mean	0.29	0.44	0.27	0.31	0.36	0.33	0.38	0.50	0.12
SD	0.09	0.10	0.12	0.04	0.04	0.06	0.09	0.10	0.07
N	119	119	119	119	119	119	119	119	119
Significance l	0.05	0.05	0.05	0.05	0.05	0.05	0.05	0.05	0.05
CI	0.28 - 0.31	0.42 - 0.46	0.24 - 0.29	0.3 - 0.32	0.35 - 0.37	0.32 - 0.34	0.36 - 0.4	0.48 - 0.51	0.11 - 0.14
Range	0.03	0.04	0.04	0.02	0.02	0.02	0.03	0.04	0.02

Patient 3 higher quality sperm (Outlier infertile patient)

	Ace Tub PC	Ace Tub DC	Ace Tub Ax	Tub PC	Tub DC	Tub Ax	POC1B PC	POC1B DC	POC1B Ax
Mean	0.39	0.49	0.13	0.40	0.42	0.18	0.37	0.52	0.11
SD	0.11	0.11	0.08	0.11	0.09	0.08	0.09	0.09	0.06
N	120	120	120	120	120	120	120	120	120
Significance l	0.05	0.05	0.05	0.05	0.05	0.05	0.05	0.05	0.05
CI	0.37 - 0.41	0.47 - 0.51	0.11 - 0.14	0.38 - 0.42	0.41 - 0.44	0.17 - 0.19	0.35 - 0.38	0.51 - 0.54	0.1 - 0.12
Range	0.04	0.04	0.03	0.04	0.03	0.03	0.03	0.03	0.02

Patient 3 lower quality sperm (Outlier infertile patient)

	Ace Tub PC	Ace Tub DC	Ace Tub Ax	Tub PC	Tub DC	Tub Ax	POC1B PC	POC1B DC	POC1B Ax
Mean	0.44	0.46	0.10	0.43	0.39	0.18	0.38	0.51	0.12
SD	0.12	0.13	0.09	0.10	0.09	0.07	0.09	0.09	0.07
N	109	109	109	109	109	109	109	109	109
Significance l	0.05	0.05	0.05	0.05	0.05	0.05	0.05	0.05	0.05
CI	0.42 - 0.46	0.44 - 0.48	0.08 - 0.12	0.41 - 0.45	0.37 - 0.41	0.17 - 0.19	0.36 - 0.39	0.49 - 0.52	0.1 - 0.13
Range	0.04	0.05	0.03	0.04	0.03	0.03	0.03	0.03	0.03

Supplemental Table 2: Most of the reference population mean FRAC ratio values had approximately Gaussian distributions.

Rows 3–12 contain the mean FRAC ratios of the three biomarkers (acetylated tubulin, tubulin, and POC1B) at each of three locations (PC, DC, and Ax) in fertile men. Rows 13 and 14 provide the predicted skewness and kurtosis for every 10 mean ratios in the reference population. Rows 15–19 provide the P-values of various normality tests. A low P-value indicates that the distribution is either non-normal or that the IID assumption was not holding. Green highlights mark approximately Gaussian distributions**.** Red highlights mark distributions that are not Gaussian**.**

	A	B	C	D	E	F	G	H	I	J
1		Acetylated Tubulin			Tubulin			POC1B		
2		PC	DC	AX	PC	DC	AX	PC	DC	AX
3	Fertile man mean FRAC ratios	0.36	0.49	0.15	0.41	0.38	0.21	0.35	0.57	0.08
4	Fertile man mean FRAC ratios	0.38	0.44	0.19	0.35	0.37	0.28	0.35	0.44	0.21
5	Fertile man mean FRAC ratios	0.36	0.45	0.19	0.36	0.41	0.23	0.35	0.48	0.16
6	Fertile man mean FRAC ratios	0.36	0.44	0.20	0.33	0.36	0.31	0.39	0.46	0.15
7	Fertile man mean FRAC ratios	0.37	0.44	0.19	0.36	0.38	0.26	0.40	0.51	0.09
8	Fertile man mean FRAC ratios	0.35	0.41	0.23	0.35	0.35	0.30	0.42	0.50	0.08
9	Fertile man mean FRAC ratios	0.36	0.46	0.18	0.34	0.39	0.27	0.34	0.50	0.16
10	Fertile man mean FRAC ratios	0.38	0.42	0.20	0.35	0.36	0.29	0.39	0.49	0.12
11	Fertile man mean FRAC ratios	0.37	0.49	0.14	0.40	0.38	0.22	0.34	0.55	0.11
12	Fertile man mean FRAC ratios	0.37	0.41	0.22	0.35	0.36	0.29	0.40	0.51	0.10
13	Skewness (normal is +/-1)	0.11	0.47	-0.26	1.12	0.65	-0.43	0.23	0.31	0.67
14	Kurtosis (normal is +/-3)	-0.65	-0.96	-0.10	0.56	0.11	-1.20	-1.55	0.22	-0.38
15	Shapiro-Wilk p-value (normal is >0.05)	0.25	0.24	0.51	0.03	0.49	0.35	0.07	0.87	0.30
16	D'Agostino-Pearson p-value (normal is >0.05)	0.93	0.72	0.73	0.13	0.48	0.46	0.19	0.82	0.54
17	Jarque-Bera p-value (normal is >0.05)	0.84	0.73	0.82	0.38	0.72	0.62	0.59	0.90	0.66
18	Cramer-von Mises p-value (normal is >0.05)	0.11	0.37	0.25	0.01	0.35	0.26	0.03	0.53	0.34
19	Anderson-Darling p-value (normal is >0.05)	0.11	0.27	0.30	0.01	0.35	0.27	0.03	0.58	0.32
20	Normality Test Calculator at https://www.gigacalculator.com/calculators/normality-test-calculator.php									

Supplemental Table 3: The reference population mean FRAC ratio values had a relatively small range.

	A	B	C	D	E	F	G	H	I	J	K
1			Fertile men - Higher quality sperm								
2				Ace Tubulin			Tubulin			POC1B	
3			PC	DC	Ax	PC	DC	Ax	PC	DC	Ax
4	Range	Max	0.38	0.49	0.22	0.41	0.41	0.3	0.42	0.57	0.21
5		Min	0.35	0.41	0.14	0.33	0.35	0.21	0.34	0.44	0.08
6		Total	0.03	0.08	0.08	0.08	0.06	0.09	0.08	0.13	0.13
7		Average		0.06			0.08			0.11	
8		SD		0.03			0.02			0.03	
10	T-test reletive to Ace tubulin	Ace Tub					0.52			0.10	
11		Tubulin								0.12	
12											
13		Eumorphic infertile men - Higher quality sperm									
14							Tubulin			POC1B	
15	Range	Total				0.08	0.09	0.12	0.14	0.21	0.2
16		Average					0.10			0.18	
17		SD					0.02			0.04	
18											
19	T-test: Healthy x eumorphic						0.25			0.06	

Supplemental Table 4: The FRAC ratio standard deviation for PC tubulin is different between fertile higher quality sperm and fertile lower quality sperm, infertile higher quality sperm, or infertile lower quality sperm.

	A	B	C	D	E	F	G	H	I	J	K	L	M	N	O	P	Q	R	S	T	U	V	W	X	Y	Z	AA
1				Higher quality sperm												Lower quality sperm											
2				Outliers number			Ace Tubulin			Tubulin			POC1B			Outliers number			Ace Tubulin			Tubulin			POC1B		
3		Patient	H+U	Total	1-SD	2-SD	PC	DC	Ax	PC	DC	Ax	PC	DC	Ax	Total	1-SD	2-SD	PC	DC	Ax	PC	DC	Ax	PC	DC	Ax
4	Fertile	10	0	0	0	0	0.36	0.49	0.15	0.41	0.38	0.21	0.35	0.57	0.08	0	0	0	0.35	0.50	0.14	0.40	0.36	0.24	0.31	0.60	0.09
5		19	0	0	0	0	0.38	0.44	0.19	0.35	0.37	0.28	0.35	0.44	0.21	0	0	0	0.38	0.44	0.18	0.35	0.36	0.29	0.35	0.46	0.20
6		2	0	0	0	0	0.36	0.45	0.19	0.36	0.41	0.23	0.35	0.48	0.16	0	0	0	0.38	0.44	0.18	0.36	0.37	0.26	0.35	0.50	0.16
7		8	0	0	0	0	0.36	0.44	0.20	0.33	0.36	0.31	0.39	0.46	0.15	0	0	0	0.36	0.44	0.20	0.34	0.36	0.30	0.40	0.47	0.13
8		1	0	0	0	0	0.37	0.44	0.19	0.36	0.38	0.26	0.40	0.51	0.09	0	0	0	0.36	0.47	0.17	0.39	0.40	0.21	0.39	0.55	0.07
9		14	0	0	0	0	0.35	0.41	0.23	0.35	0.35	0.30	0.42	0.50	0.08	0	0	0	0.36	0.40	0.24	0.39	0.36	0.25	0.39	0.49	0.12
10		7	0	0	0	0	0.36	0.46	0.18	0.34	0.39	0.27	0.34	0.50	0.16	0	0	0	0.35	0.44	0.21	0.38	0.38	0.23	0.36	0.49	0.16
11		18	0	0	0	0	0.38	0.42	0.20	0.35	0.36	0.29	0.39	0.49	0.12	0	0	0	0.40	0.42	0.18	0.33	0.35	0.32	0.37	0.51	0.12
12		4	0	0	0	0	0.37	0.49	0.14	0.40	0.38	0.22	0.34	0.55	0.11	0	0	0	0.34	0.46	0.20	0.41	0.36	0.22	0.34	0.53	0.13
13		12	0	0	0	0	0.37	0.41	0.22	0.35	0.36	0.29	0.40	0.51	0.10	0	0	0	0.32	0.46	0.22	0.32	0.37	0.31	0.37	0.52	0.11
14	Unexplained inferility	20	0	0	0	0	0.37	0.39	0.24	0.32	0.34	0.33	0.40	0.49	0.10	0	0	0	0.37	0.46	0.17	0.33	0.37	0.30	0.38	0.53	0.09
15		5	0	0	0	0	0.35	0.46	0.18	0.33	0.37	0.30	0.39	0.49	0.12	0	0	0	0.38	0.45	0.18	0.34	0.35	0.30	0.43	0.44	0.13
16		13	0	0	0	0	0.38	0.40	0.22	0.37	0.36	0.27	0.39	0.47	0.13	0	0	0	0.40	0.43	0.17	0.40	0.37	0.23	0.40	0.51	0.09
17		9	0	0	0	0	0.37	0.43	0.19	0.33	0.36	0.31	0.36	0.42	0.22	0	0	0	0.37	0.43	0.20	0.36	0.36	0.29	0.34	0.47	0.20
18		17	0	0	0	0	0.33	0.43	0.24	0.33	0.36	0.31	0.39	0.46	0.16	0	0	0	0.33	0.45	0.22	0.33	0.36	0.31	0.38	0.50	0.12
19		11	1	0	0	0	0.33	0.45	0.22	0.32	0.38	0.30	0.38	0.47	0.15	1	1	0	**0.32**	0.48	0.21	0.33	0.36	0.31	0.36	0.53	0.11
20		6	3	1	0	1	**0.30**	0.47	0.23	0.30	0.40	0.30	0.37	0.48	0.15	2	1	1	**0.28**	0.49	0.24	0.34	0.43	0.23	0.35	0.52	0.14
21		15	0	0	0	0	0.34	0.41	**0.26**	0.36	0.37	0.26	0.39	0.47	0.14	0	0	0	0.34	0.43	0.22	0.38	0.36	0.27	0.39	0.49	0.12
22		16	3	2	1	1	**0.30**	0.41	**0.29**	0.32	0.37	0.31	0.36	0.46	0.17	1	0	1	**0.29**	0.44	0.27	0.31	0.36	0.33	0.38	0.50	0.12
23		3	4	1	0	1	0.39	0.49	0.13	0.40	0.42	0.18	0.37	0.52	0.11	3	2	1	**0.44**	0.46	0.10	0.43	0.39	0.18	0.38	0.51	0.12
24	Fertile average						0.37	0.45	0.19	0.36	0.37	0.27	0.37	0.50	0.13				0.36	0.45	0.19	0.37	0.37	0.26	0.36	0.51	0.13
25						SD	0.01	0.03	0.03	0.02	0.02	0.03	0.03	0.04	0.04				0.02	0.03	0.03	0.03	0.01	0.04	0.03	0.04	0.04
26	Unexplained infertile average						0.35	0.43	0.22	0.34	0.37	0.29	0.38	0.47	0.14				0.35	0.45	0.20	0.36	0.37	0.27	0.38	0.50	0.12
28	T test pellet ankits's fertile ver infertile						0.063	0.437	0.072	0.124	0.817	0.237	0.540	0.089	0.309				0.595	0.697	0.768	0.417	0.673	0.651	0.179	0.475	0.736
29	F-test (or variance ratio test) fertile ver infertile						0.0003	0.588	0.182	0.708	0.383	0.557	0.029	0.241	0.449				0.024	0.230	0.124	0.714	0.146	0.559	0.994	0.213	0.545
30	F-test (or variance ratio test) fertile pellet ver fertile interface						0.005	0.939	0.907	0.440	0.666	0.731	0.746	0.815	0.639												
31	F-test (or variance ratio test) fertile pellet ver infertile interface						0.000	0.260	0.153	0.258	0.298	0.356	0.752	0.307	0.287												
32																											
33		Ace Tubulin Pc						Av	Sd		F test	0.0052	0.0003	6.81E-06	0.2648	0.0236	0.2193										
34		Fertile Higher quality sperm (Pellet)						0.37	0.01																		
35		Fertile Lower quality sperm (Interface)						0.36	0.02																		
36		Infertile Higher quality sperm (Pellet)						0.35	0.03																		
37		Inertile Lower quality sperm (Interface)						0.35	0.05																		
